# Patterning of the Dorsal-Ventral Axis in Echinoderms: Insights into the Evolution of the BMP-Chordin Signaling Network

**DOI:** 10.1371/journal.pbio.1000248

**Published:** 2009-11-24

**Authors:** François Lapraz, Lydia Besnardeau, Thierry Lepage

**Affiliations:** UPMC (University of Paris 06), CNRS, UMR7009, Biologie du Développement, Observatoire Océanologique, Villefranche-sur-Mer, France; Osaka University, Japan

## Abstract

Formation of the dorsal-ventral axis of the sea urchin embryo relies on cell interactions initiated by the TGFβ Nodal. Intriguingly, although *nodal* expression is restricted to the ventral side of the embryo, Nodal function is required for specification of both the ventral and the dorsal territories and is able to restore both ventral and dorsal regions in *nodal* morpholino injected embryos. The molecular basis for the long-range organizing activity of Nodal is not understood. In this paper, we provide evidence that the long-range organizing activity of Nodal is assured by a relay molecule synthesized in the ventral ectoderm, then translocated to the opposite side of the embryo. We identified this relay molecule as BMP2/4 based on the following arguments. First, blocking BMP2/4 function eliminated the long-range organizing activity of an activated Nodal receptor in an axis rescue assay. Second, we demonstrate that BMP2/4 and the corresponding type I receptor Alk3/6 functions are both essential for specification of the dorsal region of the embryo. Third, using anti-phospho-Smad1/5/8 immunostaining, we show that, despite its ventral transcription, the BMP2/4 ligand triggers receptor mediated signaling exclusively on the dorsal side of the embryo, one of the most extreme cases of BMP translocation described so far. We further report that the pattern of pSmad1/5/8 is graded along the dorsal-ventral axis and that two BMP2/4 target genes are expressed in nested patterns centered on the region with highest levels of pSmad1/5/8, strongly suggesting that BMP2/4 is acting as a morphogen. We also describe the very unusual ventral co-expression of *chordin* and *bmp2/4* downstream of Nodal and demonstrate that Chordin is largely responsible for the spatial restriction of BMP2/4 signaling to the dorsal side. Thus, unlike in most organisms, in the sea urchin, a single ventral signaling centre is responsible for induction of ventral and dorsal cell fates. Finally, we show that Chordin may not be required for long-range diffusion of BMP2/4, describe a striking dorsal-ventral asymmetry in the expression of Glypican 5, a heparin sulphated proteoglycan that regulates BMP mobility, and show that this asymmetry depends on BMP2/4 signaling. Our study provides new insights into the mechanisms by which positional information is established along the dorsal-ventral axis of the sea urchin embryo, and more generally on how a BMP morphogen gradient is established in a multicellular embryo. From an evolutionary point of view, it highlights that although the genes used for dorsal-ventral patterning are highly conserved in bilateria, there are considerable variations, even among deuterostomes, in the manner these genes are used to shape a BMP morphogen gradient.

## Abstract 2

Deciphering the process of dorsal-ventral patterning in the sea urchin reveals an extreme case of BMP translocation and an unusual configuration of the BMP-Chordin axis in echinoderms.

## Author Summary

During early development of many organisms, patterning along the dorsal-ventral axis is regulated by the activities of two signaling centers located on the ventral and dorsal sides of the embryo. One of these centers produces growth factors of the BMP family that act as morphogens, whereas the other center secretes BMP antagonists such as Chordin that regulate the flow of BMPs along the dorsal-ventral axis. Expression from these two signaling centers results in roughly complementary distributions of BMP and BMP antagonist. We have analyzed BMP-mediated dorsal-ventral axis patterning in embryos of sea urchins, which are phylogenetically close to vertebrates and extensively rely on cell-cell interactions for their development. We found that in sea urchins, unlike in most organisms, the activity of a single signaling center located on the ventral side is responsible for generating both the ventral and the dorsal sides of the embryo. In addition, we discovered that the BMP2/4 gene is co-expressed with Chordin in this ventral center but that the BMP2/4 protein is translocated to the opposite side of the embryo where it activates the genetic program responsible for dorsal differentiation. Our study reveals an unusual example of signaling at a distance by a BMP growth factor. It also highlights that although the proteins used for dorsal-ventral patterning are evolutionarily conserved, there are considerable variations in the manner in which these proteins can be used in different species to generate a gradient of BMP morphogen.

## Introduction

Genetic and molecular studies carried out in vertebrates and invertebrates have shown that dorsal-ventral (D/V) patterning in bilaterians is regulated by a remarkably conserved patterning system which relies on production of secreted BMP inhibitors such as Chordin (Sog in *Drosophila*) which antagonize the activity of BMP signals (Dpp in *Drosophila*) resulting in a gradient of BMP activity along the D/V axis [1]–[4]. Accordingly, in all the bilaterians where expression of *chordin* and *BMP2/4* has been analyzed, these genes show complementary expression in opposite territories along the D/V axis leading to the important concept that dorsal and ventral cells communicate by signals emanating from dorsal and ventral signaling centers [5]. Intriguingly, although the function of these genes has been conserved between vertebrates and invertebrates, their expression pattern along the D/V axis are inverted, such that *dpp* is expressed dorsally in invertebrates while *BMP2/4* is expressed ventrally in vertebrates, suggesting that an inversion of the D/V axis has occurred in the course of evolution [6],[7].

**Figure 1:**
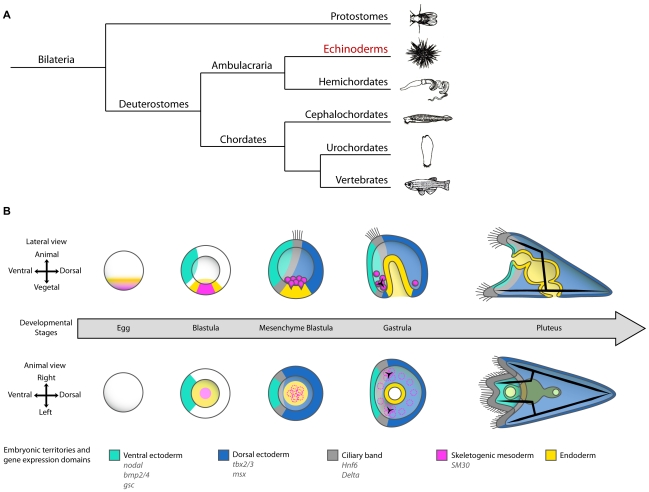
Position of echinoderms in phylogeny of bilateria and establishment of D/V polarity during early development of the sea urchin embryo. (A) Simplified phylogenetic tree of bilateria. Echinoderms together with hemichordates form a sister group of the chordates. (B) Scheme describing the early development of the sea urchin embryo together with the fate maps and gene expression territories. D/V polarity first becomes apparent at the beginning of gastrulation by the flattening of the presumptive ventral side, the bending of the gut, and the asymmetrical positioning of the skeletogenic mesenchyme cells that build the spicules. D/V polarity is further accentuated when four arms grow on the ventral side while the mouth opens on the ventral ectoderm and the larva lengthens along the dorsal side to become the pluteus larva.

Echinoderms offer interesting models for the comparative analysis of the mechanisms of axis specification. Their position at the basis of the deuterostome lineage makes them a valuable model to reconstruct the evolution of deuterostomes from an ancestral metazoan and to understand how the chordate body plan emerged (Figure 1A). One peculiar feature of echinoderms is that most of them develop indirectly, i.e. the adult, which emerges through a metamorphosis and displays very little similarity with the larva, is built from groups of cells that are set aside during embryogenesis [8]. Although the adult body plan of echinoderms is typically radial, this feature is a recent modification of a bilateral body plan and echinoderm larvae are indeed bilaterally symmetrical as are larvae of their closest relatives, the hemichordates, which undergo no axial remodeling. Indeed, it has been recognized for a long time that larvae of indirectly developing echinoderms and larvae from directly developing hemichordates share many morphological features [9]. It is therefore most likely that despite considerable differences in their adult body plans and mode of development, these homologous larval features of echinoderms and hemichordates reflect utilization of similar genes and gene regulatory mechanisms during embryogenesis. Since the ambulacraria form a sister group of the chordates, it is also reasonable to postulate that the genes, transcription factors, and signaling pathways that orchestrate embryonic axial patterning in chordates may have a conserved role and echinoderms. This idea is largely supported by recent findings showing that echinoderms use many of the same regulatory genes and signaling pathways employed by more complex bilateria. The conserved role of the Wnt/beta catenin in animal vegetal patterning [10],[11] as well as the conserved role of the Nodal/Univin/vg1 in D/V patterning [12],[13] and in left right axis determination [14] even suggests a conservation at the wider gene regulatory network level.

The D/V polarity of the sea urchin larva first becomes morphologically apparent at the early gastrula stage when bilateral clusters of skeletogenic mesenchyme cells form on the presumptive ventral side (Figure 1B). Later during gastrulation, the larva flattens on the presumptive ventral side and the archenteron bends towards the presumptive ventral ectoderm where the mouth will open. As morphogenesis continues, D/V polarity becomes even more prominent with the larva elongating along the D/V axis and acquiring its characteristic easel-like shape. At the pluteus stage, the ectoderm is divided into two main territories: the ventral (or oral) ectoderm and the dorsal (or aboral) ectoderm separated by a belt of cuboidal ciliated cells and neurons, the ciliary band (Figure 1B).

Classical studies revealed that D/V axis formation in sea urchin embryos extensively relies on cell interactions [15]. In one of the most famous experiments of embryology, Hans Driesch demonstrated that after isolation, each of the four blastomeres of a four-cell stage embryo can give rise to a normal pluteus larva with a perfectly normal D/V axis. Not only did this experiment demonstrate the astonishing regulative capacity of sea urchin embryos, but it also showed that the D/V axis is not fixed in the early embryo and that formation of this axis must therefore rely on cellular communication.

Recent studies have shown that the TGF-β ligands Nodal, Vg1/Univin, and BMP2/4 all play crucial roles in these cell interactions [12],[13],[16],[17]. Expression of *nodal* is initiated around the 32–60-cell stage in most cells of the presumptive ectoderm and is rapidly restricted to the presumptive ventral ectoderm. *nodal* is the earliest zygotic gene found to display a restricted expression along the D/V axis during sea urchin development, and Nodal function appears to be required for all aspects of D/V polarity of the embryos [16]. When translation of the Nodal transcript is prevented, specification of both the ventral and the dorsal ectoderm fails and most of the ectoderm (except the ectoderm derived from the animal pole and vegetal pole regions) differentiates into a thickened ciliated ectoderm that shares features with the neurogenic territory of the ciliary band. The ciliary band may thus represent the default fate of most of the ectoderm in the absence of Nodal [16]. Functional studies have shown that overexpression of Nodal causes most ectodermal cells to adopt a ventral fate. Even more strikingly, injection of synthetic *nodal* mRNA into one blastomere at the eight-cell stage is sufficient to fully rescue axis formation in *nodal* morpholino injected embryos. It is thus clear that Nodal expressing cells have a long-range organizing activity and are capable of restoring D/V polarity over the whole embryo [14],[16]. Consistent with these observations, sea urchin Nodal activates a regulatory network of genes encoding key transcription factors such as FoxA, Goosecoid, and Brachyury as well as signaling molecules such as BMP2/4 and the Nodal antagonist Lefty, which restricts Nodal activity [12],[14],[18].

Although the organizing activity of Nodal expressing ventral cells is the key to D/V patterning in the sea urchin, the nature of the long-range signal that specifies the dorsal territory is not understood. Theoretically, the non-autonomous effect of Nodal could either be direct, with Nodal working as a long-range morphogen, or indirect, via induction of a second relay molecule. A direct role for Nodal is supported by previous studies in zebrafish and *Xenopus* demonstrating that secreted Nodal and Activin ligands can act as morphogens and diffuse over long distances to specify different cell fates [19],[20]. It is thus possible that in the sea urchin embryo, Nodal could diffuse from its site of production and specify the ventral and dorsal ectoderm territories in a dose-dependent manner. Various observations indicate, however, that Nodal may not act directly to specify dorsal cell fates but may induce production of a diffusible relay molecule. First, it has been shown that the range of action of Nodal ligands is limited by the activity of Lefty antagonists [18],[21]–[25]. Second, studies on the inhibitory effects of Nodal on specification of neural fates at the animal pole of the embryo have indicated that the effects of Nodal are short range and restricted to only a few cell rows away from the Nodal expressing territory [26],[27]. Finally, an attractive candidate for a relay molecule exists in BMP2/4 [16],[17]. BMP2/4 is expressed zygotically downstream of Nodal signaling from the 128-cell stage to the prism stage, with its transcripts detected exclusively on the presumptive ventral side of the embryo. Despite being ventrally expressed, BMP2/4 appears to be required on the opposite side of the embryo for expression of dorsal marker genes such as *tbx2/3* and the novel transmembrane protein 29D [16]. Although BMP2/4 is thus an appealing candidate for a relay molecule downstream of Nodal, this hypothesis raises important issues concerning the evolution of the D/V patterning system in bilateria, since both Dpp in *Drosophila* and BMP2/4 in vertebrates act on the side of the embryo where they are expressed and not on the opposite side as predicted in the sea urchin.

In this study, we have analyzed the mechanism by which Nodal and BMP2/4 ligands pattern the D/V axis of the sea urchin embryo. We provide evidence that Nodal does not work as a long-range morphogen along the D/V axis but requires a relay molecule that we confirmed as BMP2/4. We first show that microinjection of an activated form of the Nodal receptor non-autonomously rescues dorsal structures in *nodal* morpholino injected embryo by inducing BMP signaling in cells located on the opposite side of the embryo. Second, by using a phospho-Smad immunostaining, we showed that, although BMP2/4 is expressed on the ventral side, BMP2/4 signaling is active exclusively on the dorsal side of the embryo, far away from its site of production. Third, we showed that the functions of BMP2/4 and of its putative receptor Alk3/6 are not required for specification of ventral cell fates but are essential for specification of dorsal cell fates. In addition, we have examined the expression and function of sea urchin Chordin and found it to be necessary to prevent BMP signaling on the ventral side. In contrast with the situation in other bilaterian models, however, the territories expressing *chordin* and BMP2/4 are not complementary but are congruent. Thus, although sea urchin embryos have a conserved BMP-Chordin axis, it is manifest by the activity of these molecules but not their site of production. Finally, we report that Chordin may not be required for diffusibility of BMP2/4 and that BMP2/4 signaling induces a positive feedback mechanism in responsive cells by inducing the expression of *glypican 5*, a positive regulator of BMP signaling and mobility. Based on our detailed dissection of D/V patterning in the sea urchin embryo, we propose a new model and discuss the evolutionary implications of these findings.

## Results

### Nodal Does Not Act Directly as a Morphogen to Pattern the D/V Axis But Requires BMP2/4

**Figure 2:**
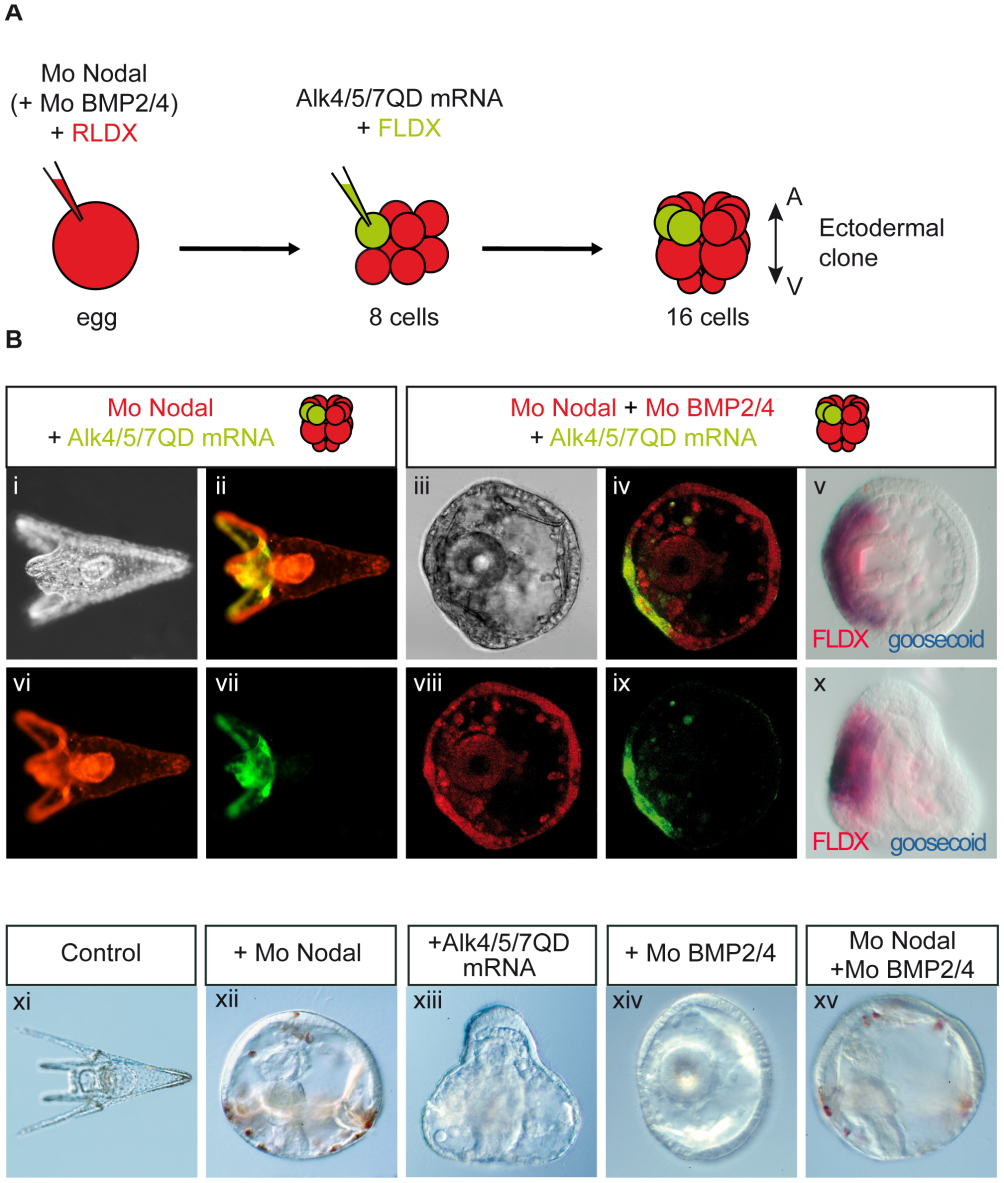
Rescue of the D/V axis of *nodal* morpholino injected embryos by an activated Nodal receptor requires BMP2/4. (A) Scheme of the experiment. Eggs were injected with a morpholino directed against *nodal* together with a red fluorescent dextran (RLDX). At the eight-cell stage, embryos were then re-injected into one randomly chosen blastomere with the *alk4/5/7* mRNA together with a green fluorescent dextran (FLDX) as a lineage tracer. At the 16-cell stage, embryos were sorted according to the type of blastomere targeted by the injection (animal or vegetal) and allowed to develop in separate dishes. Fluorescent and DIC observations were made 72 h after fertilization. Only embryos derived from injection into an animal blastomere are presented here. See Figure S1 for the results of vegetal blastomere injections. (B) (i, ii, vi, vii) Typical morphology of a 48 h rescued embryo derived from injection of *alk4/5/7QD* mRNA into an animal blastomere. Although development of these embryos is slightly delayed, they display a perfectly normal D/V axis (i) and look like control embryos (xi). (iii–v, viii–x) injection of *alk4/5/7QD* mRNA in an animal blastomere at the eight-cell stage fails to restore the D/V axis of embryos previously co-injected with the *nodal* and *BMP2/4* morpholinos. These embryos never elongate and look like *BMP2/4* morpholino injected embryos (xiv). In these embryos, all cells inherited the morpholino(s), as evidenced by the RLDX red fluorescence (viii). Although the dorsal side is not rescued, the ventral ectoderm is specified as indicated by the expression of *goosecoid* in the clone of *Alk4/5/7QD* injected cells (v,x) (100%, *n* = 17). In (v, x) presence of the FLDX lineage tracer was revealed using an anti-fluorescein antibody conjugated to alkaline phosphatase and using Fast red as substrate. Therefore, the lineage tracer appears in red. Embryos injected simultaneously with the *nodal* and *BMP2/4* morpholinos (xv) are radialized and indistinguishable from embryos injected with the *nodal* morpholino alone (xii). (ii, vi, vii) are animal views observed under an optical fluorescence microscope. (iv, viii, ix) are latitudinal confocal optical sections of a representative embryo. Red (vi, viii): RLDX; green (vii,ix): FLDX, (ii, iv) show overlays.

In order to test whether Nodal works as a morphogen along the D/V axis or through a relay molecule, we used an axis rescue assay [16]. We showed previously that microinjection of synthetic mRNA encoding the secreted Nodal ligand into one animal blastomere at the eight-cell stage fully rescues the D/V axis of embryos pre-injected with an antisense *nodal* morpholino oligonucleotide at the egg stage. To determine whether a long-range non-autonomous activity of Nodal is required for the rescue to be effective, we made use of the sea urchin Nodal receptor Alk4/5/7 [13]. To activate Nodal signaling cell autonomously, we constructed an activated form of this receptor by following a strategy similar to that used to make an activated version of the zebrafish Nodal receptor TARAMA, mutating the glutamine residue found in position 265 into an aspartic acid (see Figure S1–S3) [28] and tested if injection of mRNA encoding this activated Nodal receptor into an animal blastomere at the eight-cell stage was able to rescue the radialized phenotype of *nodal* morphants (Figure 2A). Strikingly, all embryos (*n*>50) that received the *alk4/5/7QD* mRNA into an animal blastomere developed into normal pluteus larvae with a harmoniously patterned D/V axis indicating that the activated Nodal receptor was just as efficient as the Nodal ligand in this rescue experiment (Figure 2Bi, ii, vi, vii). Even embryos injected into a vegetal blastomere that, according to the fate map, will contribute to the endomesoderm as well as to the most vegetal part of the ectoderm were rescued to a considerable extent (Figure S1). As observed in the case of the rescue with the Nodal ligand, the progeny of the blastomere injected with *alk4/5/7QD* mRNA was always found on the ventral face of the rescued pluteus larvae, consistent with the idea that Nodal signaling promotes ventral fates (Figure 2Bii, vii). More importantly, this result indicates that Nodal signaling is not required outside the ventral ectoderm to specify the dorsal ectodermal fates, implying that Nodal induces a relay molecule that is in turn responsible for specification of the dorsal ectoderm.

We then tested if BMP2/4 function is required for the rescue of dorsal structures by ectopic activation of the Nodal pathway in *nodal* morphants (Figure 2Biii–v, viii–x). Embryos were injected with both *nodal* and *BMP2/4* morpholinos at the one-cell stage and *alk4/5/7QD* was injected into one blastomere at the eight-cell stage. Control embryos injected with a mixture of the *BMP2/4* morpholino and the *nodal* morpholino were radialized and undistinguishable from embryos injected with the *nodal* morpholino alone (Figure 2Bxii–xv) consistent with BMP2/4 being a downstream target of Nodal. Injection of *alk4/5/7QD* into these double-morpholino embryos failed to rescue a full D/V axis, producing embryos that were polarized but that failed to elongate on the dorsal side (Figure 2Biii, iv, viii, ix). The arms of the pluteus larva failed to form, however the ventral ectoderm was specified in these embryos as shown by the expression of *goosecoid* in the clone of injected cells and by the asymmetrical positioning of the gut close to the *alk4/5/7* expressing cells (Figure 2Bv, x) (*n* = 17). On the presumptive dorsal side, the epithelium remained thick, and typically, ectopic spicules were present (Figure 2Biii). These embryos resemble the *BMP2/4* morpholino injected embryos (Figure 2Bxiv). We conclude that in the double *nodal* and *BMP2/4* morpholino injected embryos, ectopic expression of *alk4/5/7QD* was able to restore ventral cell fates but failed to restore the dorsal cell fates—in other words, that BMP2/4 expression is required in the ventral ectoderm downstream of Alk4/5/7 to induce dorsal cell fates.

### BMP2/4 and a BMP Receptor Are Required for Specification of the Dorsal Ectoderm

**Figure 3:**
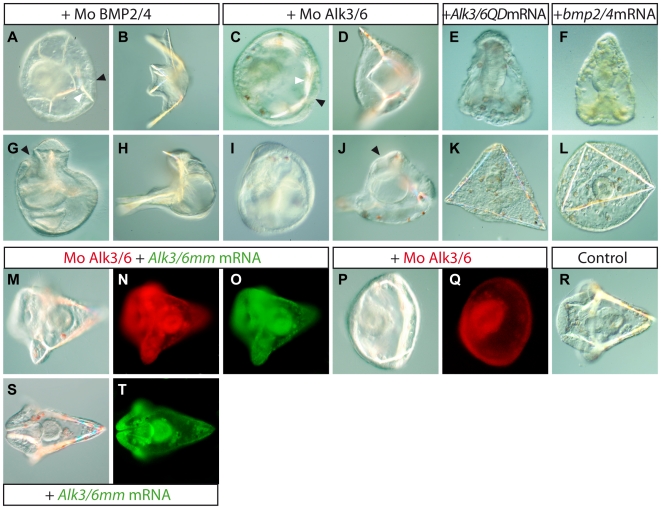
BMP2/4 and Alk3/6 are required for specification of the dorsal ectoderm. Embryos injected with morpholino oligonucleotides directed against *BMP2/4* (0.4 mM) (A, G) or *alk3/6* (0.8 mM) (C, I) are strongly radialized. They nevertheless retain D/V polarity since the gut is always positioned asymmetrically and bends towards the side of the embryo where a mouth opens (arrow in G and J). A characteristic feature of this phenotype is the presence of ectopic spicules on the side opposite to the mouth (white arrows) associated with a thickened epithelium (black arrows) that resembles the epithelium of the ciliary band (A, C). Injection of lower doses of the BMP2/4 morpholino oligonucleotide (0.25 mM) appeared to affect preferentially development of the dorsal region leaving the ventral region relatively unaffected (B, H). The resulting embryos looked like plutei but had striking truncations of the dorsal side. Similarly, injection of the *alk3/6* morpholino at 0.6 mM prevented development of the dorsal side but allowed partial development of ventral arms (D, J). Embryos overexpressing *alk3/6QD* (E, K) or *BMP2/4* (F, L) mRNA are also strongly radialized. When observed at 48 h (E, F), they show a typical elongated shape and are covered by a thin and wrinkled ectoderm characteristic of the dorsal ectoderm. Starting at 72 h, however, they contain very long unbranched spicules (K, L) reminiscent of the spicules normally found on the dorsal side in wild type embryos (R). The embryos in (A–D) are viewed from the animal pole while the embryos in (E–J) are viewed from the side. The embryos in (K, L) are viewed from the vegetal pole. (M–T) Rescue experiment. While all the embryos injected with the *alk3/6* morpholino alone developed with a radialized phenotype (P, Q), about half of the embryos injected with both the *alk3/6* morpholino and a 300 mg/ml solution of synthetic *alk3/6* mRNA containing nine mismatches in the sequence recognized by the morpholino (*alk3/6mm*) developed into pluteus larvae, like control embryos (R) and embryos injected with the *alk3/6mm* mRNA (S, T). The remaining embryos were either only partially rescued (30%) or not rescued to a significant extent.

We had previously reported that inhibition of BMP2/4 mRNA translation by morpholino injection causes embryos to develop with a severe radialized phenotype [16]. A careful re-examination of this phenotype revealed several interesting features. First, injection of low concentrations (0.25 mM) of this morpholino frequently caused embryos to develop with a truncated dorsal region (Figure 3B, 3H) leaving the ventral region relatively unaffected, while injection of higher doses (0.4 mM) resulted in severe radialization (Figure 3A, 3G) as reported previously. This suggested that specification of the dorsal most region of the embryo is highly sensitive to BMP2/4 inhibition and that the lateral region of the embryo, from which the arms emerge, is less sensitive to reductions of BMP2/4 function. Second, we noted that although all the embryos injected at 0.4 mM appeared radialized, they retained a D/V polarity as shown by the opening of the mouth and by the bending of the gut towards one side of the embryo (Figure 3A, 3G). Thus, even high doses of this morpholino do not block differentiation of the ventral ectoderm. Intriguingly, most of the ectoderm covering these embryos was made of a thick ectoderm that resembled the ciliary band of pluteus larvae and multiple ectopic spicules frequently developed in association with this thickened ectoderm (Figure 3A, 3G).

To further investigate BMP2/4 function and determine if it fulfills the requirements for a relay molecule that acts downstream of Nodal to induce dorsal fates, we cloned and characterized one of its putative receptors. The genome of *Strongylocentrotus purpuratus* contains two genes encoding type I BMP receptors, *alk3/6* and *alk1/2*, homologous to the vertebrate BMP type I receptors. Previous phylogenetic analyses indicated that Sp-Alk3/6 is mostly related to the *Drosophila* Thickveins receptor, the primary receptor for Dpp, while Sp-Alk1/2 is mostly related to *Drosophila* Saxophone, which preferentially binds Screw (Scw), a distantly related BMP ligand [29]–[35]. We isolated a cDNA encoding the *Paracentrotus lividus* Alk3/6 (Figures S2 and S3) and found that *alk3/6* transcripts are expressed maternally and ubiquitously at all stages (unpublished data). This suggests that all cells of the sea urchin embryo are likely competent to respond to BMP signaling via this receptor and that spatial and temporal restriction of these signals is most likely due to the availability of a restricted source of ligands. Embryos injected with an antisense oligonucleotide morpholino directed against the translation initiation site of the *alk3/6* transcript developed apparently normally up to the prism stage but then displayed a characteristic and highly penetrant phenotype. At 72 h, while the control embryos reached the pluteus stage, the injected embryos failed to elongate along the D/V axis and showed ectopic spicules associated with a thickened epithelium reminiscent of the ciliary band (Figure 3C, 3I). Importantly, the squamous epithelium that is normally found on the dorsal side never formed in *alk3/6* morpholino embryos suggesting that specification of the dorsal ectoderm had failed. As in the case of BMP2/4, high concentration (0.8 mM) of this morpholino caused a complete radialization while lower concentrations (0.6 mM) prevented development of the dorsal side but allowed formation of a pair of ventral arms (Figure 3D, 3J). Also, as observed in the case of *BMP2/4* morphants, these embryos retained some polarity along the D/V axis as indicated by the opening of the mouth, the asymmetric positioning of the gut on one side of the embryo, and the asymmetric morphogenesis of the ectoderm. This set of phenotypes, i.e. failure of the embryo to elongate, presence of ectopic spicules underneath a thickened ectoderm, is strikingly similar to the set of phenotypes observed following inhibition of BMP2/4 translation (see Duboc et al. 2004 [16] and, in this paper, Figure 2Bxiv and Figure 3A, 3G). To test the specificity of the *alk3/6* morpholino, we performed a rescue experiment. Eggs were first injected with the *alk3/6* morpholino, then later in the first cell cycle re-injected with a synthetic *alk3/6* mRNA containing nine mismatches (*alk3/6 mm*) in the sequence recognized by the morpholino (Figure 3M–O). While injection of the *alk3/6* morpholino caused embryos to develop with the radialized phenotype described above (Figure 3P, 3Q), half of the embryos that subsequently received injection of the *alk3/6 mm* mRNA developed with well developed dorsal arms and dorsal sides (Figure 3M, 3O).

We next constructed an activated form of the Alk3/6 receptor, using the same strategy as for the Alk4/5/7 receptor, mutating Glutamine in 230 into an Aspartic acid (Figure S2B). Embryos injected with mRNA encoding this mutant receptor (Alk3/6QD) developed with a completely radialized phenotype and lacked all D/V polarity at 48 h (Figure 3E). Except for the animal pole, where a thick proboscis formed, all the ectoderm of these embryos differentiated into a thin, squamous, and pigmented epithelium typical of the dorsal ectoderm. This phenotype is largely similar to the phenotype obtained by overexpressing BMP2/4 (Figure 3F), which was shown to reflect re-specification of most of the ectoderm into dorsal ectoderm [16]. Strikingly, starting at 72 h, long unbranched spicules strongly resembling the spicules normally found on the dorsal side of wild type embryos started to form in both the BMP2/4 and Alk3/6QD overexpressing embryos (Figure 3K, 3L). Taken together these results strongly suggest that signals transduced by the Alk3/6 receptor are required to induce dorsal ectoderm fates probably downstream of BMP2/4 binding.

**Figure 4:**
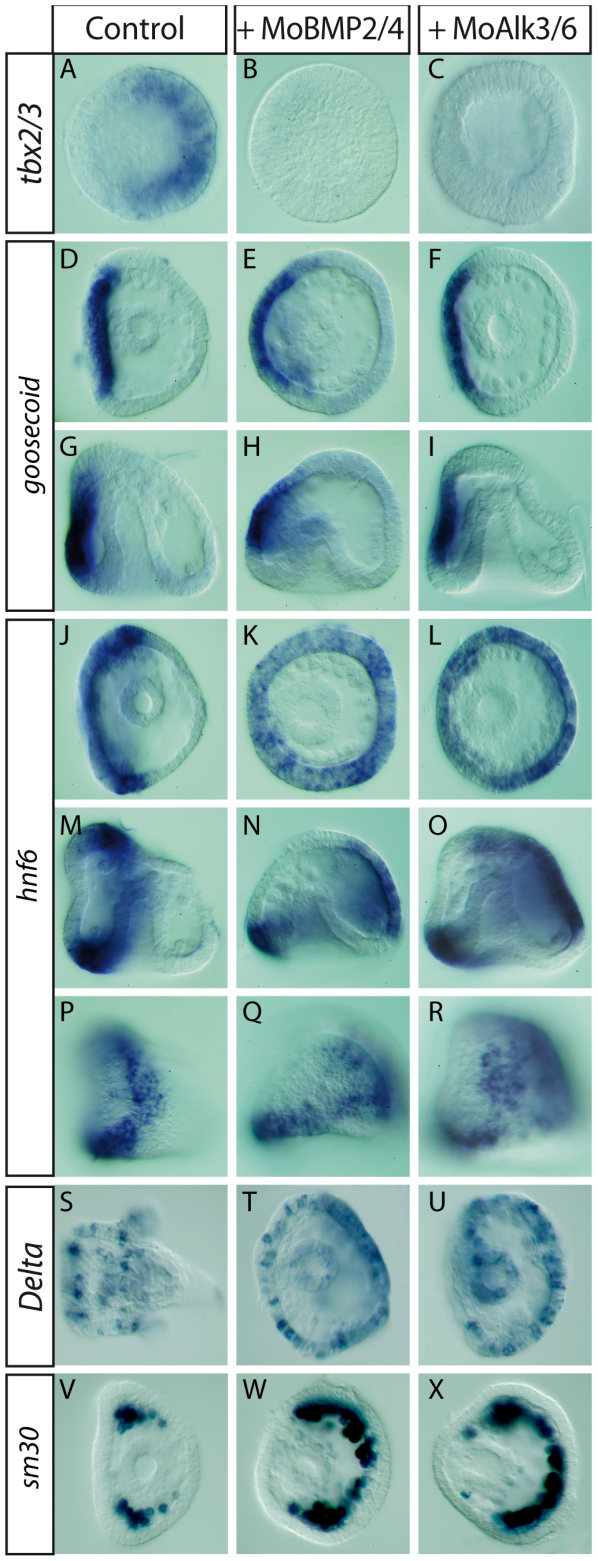
Knockdown of BMP2/4 or Alk3/6 suppresses specification of the dorsal ectoderm and expands the ciliary band territory without affecting the expression of ventral marker genes. Expression of the dorsal marker gene *tbx2/3* is abolished in the *bmp2/4* (B) or *alk3/6* (C) morphants. The *goosecoid* gene is expressed in the ventral ectoderm of control embryos (D, G). *goosecoid* expression is unaffected in *bmp2/4* (E, H) or *alk3/6* morphants (F, I). In control late gastrulae, *hnf6* expression is restricted to a belt of ectodermal cells at the boundary between the ventral and dorsal ectoderm that constitutes the presumptive neurogenic ciliary band (J, M, P). In both the *bmp2/4* (K, N, Q) or *alk3/6* (L, O, R) morpholino injected embryos, *hnf6* expression dramatically expands into the presumptive dorsal ectoderm, filling most of this territory. At 48 h, *Delta* expression labels individual neurons of the facial ectoderm and ciliary band (S). In both the *bmp2/4* and *alk3/6* morphants, strong ectopic expression of *Delta* is detected in the presumptive dorsal ectoderm. The *sm30* gene, which encodes a spicule matrix protein [82], is preferentially expressed in the bilateral clusters of skeletogenic mesenchyme cells that form at the level of the presumptive ciliary band (V) [41]. In both the *alk3/6* and *bmp2/4* morphants, clusters of skeletogenic mesenchyme cells expressing *sm30* are present under the thickened ectoderm on the presumptive dorsal side (W, X). These phenotypes are consistent with the idea that the default fate of the dorsal ectoderm in the absence of BMP signaling may be a neurogenic ciliary band-like fate. Embryos in (C, G–I, M–R) are seen from the side while embryos shown in (A, B, D–F, J–L, S–X) are seen from the vegetal pole.

To compare the phenotypes caused by inhibition of BMP2/4 and Alk3/6 at the molecular level, we examined the expression of *tbx2/3*, *goosecoid*, and *onecut/hnf6* transcripts, normally expressed in ectodermal domains corresponding, respectively, to the dorsal, ventral, and ciliary band territories (Figure 4B) [16],[36]–[40]. Blocking BMP2/4 function or Alk3/6 function abolished the expression of *tbx2/3* (Figure 4A–C) but did not affect the ventral marker gene *goosecoid* (Figure 4D–I). In contrast, expression of *onecut/hnf6*, which is expressed in the neurogenic territory of the ciliary band, was dramatically affected following inhibition of BMP signaling. Both the *alk3/6* and *BMP2/4* morpholinos caused an expansion of *onecut/hnf6* in the territory where *tbx2/3* failed to be expressed (Figure 4J–R). This prompted us to examine the expression of *Delta*, a marker gene expressed in neurons of the larva. Expression of *Delta*, which is normally restricted to individual neurons of the ciliary band and facial ectoderm, was dramatically expanded to the thickened ectoderm region facing the ventral side in the *BMP2/4* and *alk3/6* morphants (Figure 4S–U). This strongly suggested that the dorsal ectoderm adopted the fate of the ciliary band, or in other words that the dorsal ectoderm failed to form and was replaced by a more lateral ectoderm. To confirm this hypothesis, we examined the expression of the *sm30* gene, which is normally expressed in the bilateral clusters of skeletogenic mesenchymal cells that form at the level of the presumptive ciliary band (Figure 1) [41]. Indeed, *sm30* was expressed ectopically in clusters of skeletogenic cells underlying the thickened ectoderm of *BMP2/4* and *alk3/6* morphants (Figure 4V–X). This supports our previous suggestion that in the absence of Nodal, most of the ectoderm adopts a default fate and differentiates into a neurogenic ectoderm expressing gene markers of the ciliary band [16],[27]. Importantly, the phenotypes obtained by blocking BMP2/4 or Alk3/6 are indistinguishable both at the morphological and molecular level, indicating that Alk3/6 function is essential to transduce BMP2/4 signaling.

### A BMP Ligand Produced on the Ventral Side Activates the BMP Pathway on the Dorsal Side of the Embryo

**Figure 5:**
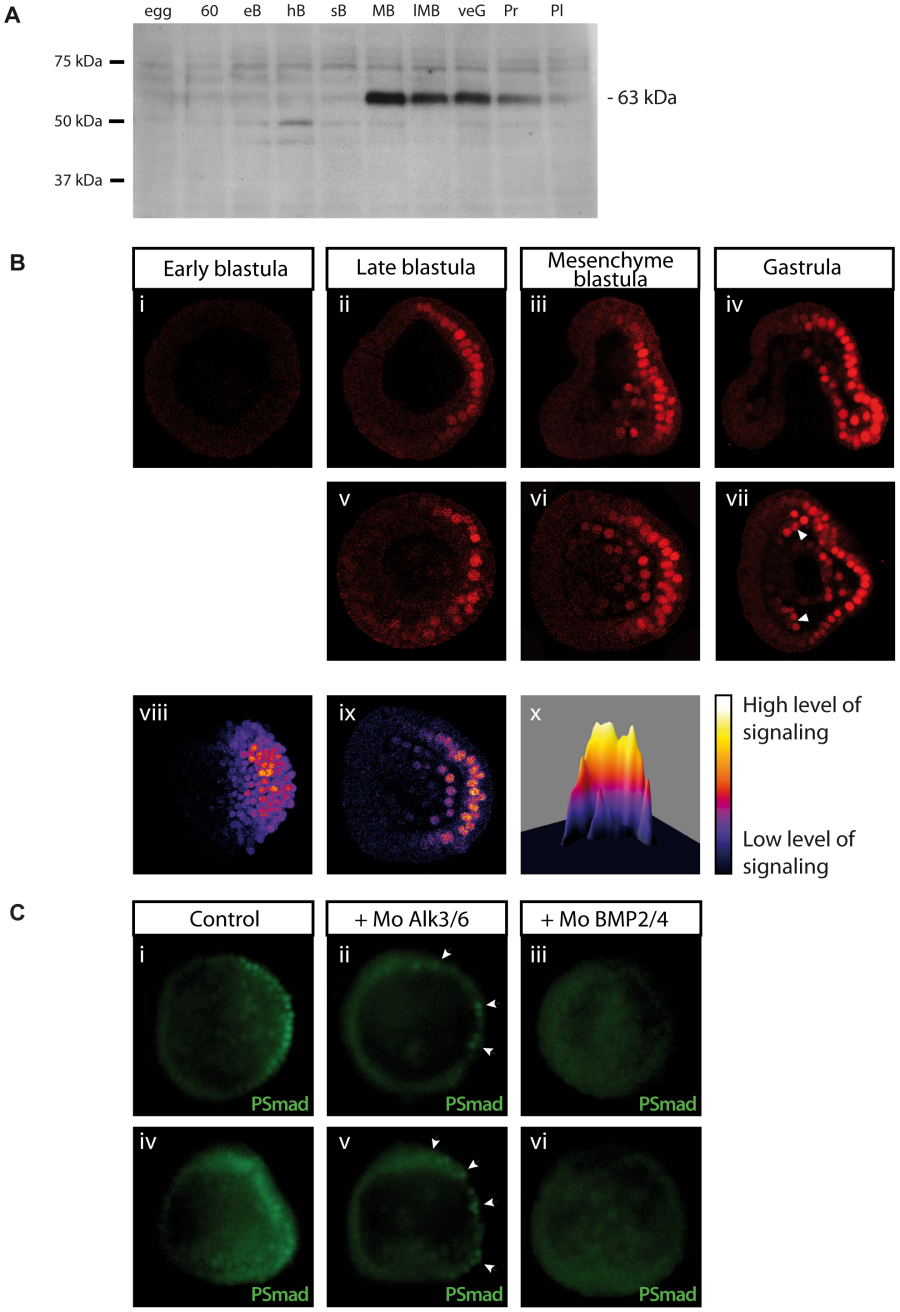
Kinetics, pattern, and BMP2/4 dependence of pSmad1/5/8 during development of the sea urchin embryo. (A) Time course analysis of pSmad1/5/8 signaling analyzed by Western blot. 60, 60-cell stage; eB, early blastula; hB, hatching blastula; sB, swimming blastula; MB, mesenchyme blastula; lMB, late mesenchyme blastula; veG, very early gastrula; Pr, prism stage; Pl, pluteus stage. The major antigen recognized by the anti-pSmad1/5/8 antibody accumulates at mesenchyme blastula and gastrula stages. (B) pSmad1/5/8 pattern during embryogenesis. The pSmad1/5/8 signal first appears at the late blastula stage, immediately before ingression of the primary mesenchyme cells, in roughly half of the embryo (i, ii, v). At mesenchyme blastula stage, strong nuclear staining is observed in half of the embryo, both in the ectoderm and in skeletogenic mesenchyme cells (iii, vi). At gastrula stages, the staining is still restricted to half of the embryo and encompasses all three germ layers (iv, vii). Note that some nuclei within the clusters of skeletogenic mesenchyme cells are labeled (white solid arrowheads). (viii) Reconstructed 3D animal view of a representative control embryo using 40 section images and using the “Volume viewer” ImageJ plugin. (ix) Representative single section image corresponding to a latitudinal section of a control embryo, dorsal side is on the right. (x) Surface plotting of the image in (ix) using the “Interactive 3D Surface Plot v2.32” ImageJ plugin. The curve is centered on the dorsal side of the embryo. All the images are colored with false colors. Low signals are colored in blue, high signals are colored in white. (C) pSmad1/5/8 staining is dependent on BMP2/4 and Alk3/6. (i, iv) Control mesenchyme blastula embryos displaying asymmetric nuclear pSmad1/5/8 immunostaining on one side of the embryo. This staining is greatly reduced in *alk3/6* morpholino injected embryos (ii, v). In these embryos only residual patches of fluorescent nuclei are detected (white arrows). In *bmp2/4* morpholino injected embryos, no staining is detectable. The embryos in (i–iii) are seen from the animal pole while the embryos in (iv–vi) are seen from the side.

The experiments described above strongly suggest that BMP2/4 acts via Alk3/6 to specify the dorsal ectoderm of the embryo. To address whether ventrally expressed BMP2/4 diffuses from the ventral side where it is synthesized and activates Alk3/6 on the opposite side of the embryo or if it induces a second relay molecule in the ventral ectoderm, we sought to determine where in the embryo the BMP pathways are active by performing an anti-phospho-Smad immunostaining. In the sea urchin, there are only two genes encoding R-Smads: Smad1/5/8 and Smad2/3 [29]. To visualize the phosphorylated form of these Smad factors, we used an antibody directed against the phosphorylated form of human Smad5. On Western blot of *P. lividus* protein extracts, this antibody recognized predominantly one protein, the abundance of which increased abruptly at the mesenchyme blastula stage (Figure 5A). By immunostaining, this antibody detected asymmetrical nuclear phospho-Smad staining in roughly one half of the embryo starting at the onset of ingression of the primary mesenchyme cells (Figure 5Bi, ii, v). Interestingly, this pSmad staining appeared graded with the highest levels being detected in the dorsal midline (Figure 5Bviii–x). This staining intensified at the mesenchyme blastula and gastrula stages with strong signaling being detected in one half of the embryo in all three germ layers (Figure 5Biii, iv, vi, vii) and peak levels in the dorsal midline. In embryos in which BMP2/4 function was blocked, this pSmad staining was totally lost (Figure 5Ciii, vi), while in the *alk3/6* morpholino injected embryos, the pSmad staining was strongly reduced, with only a residual staining being detected in patches of cells (Figure 5Cii, v). Since at the mesenchyme blastula stage *BMP2/4* morphants do express *nodal* [16], the absence of pSmad staining in the *BMP2/4* morphants together with the absence of pSmad staining at the early blastula stages in wild type embryos indicate that, in *P. lividus*, this antibody recognizes specifically the phosphorylated form of Smad1/5/8.

**Figure 6:**
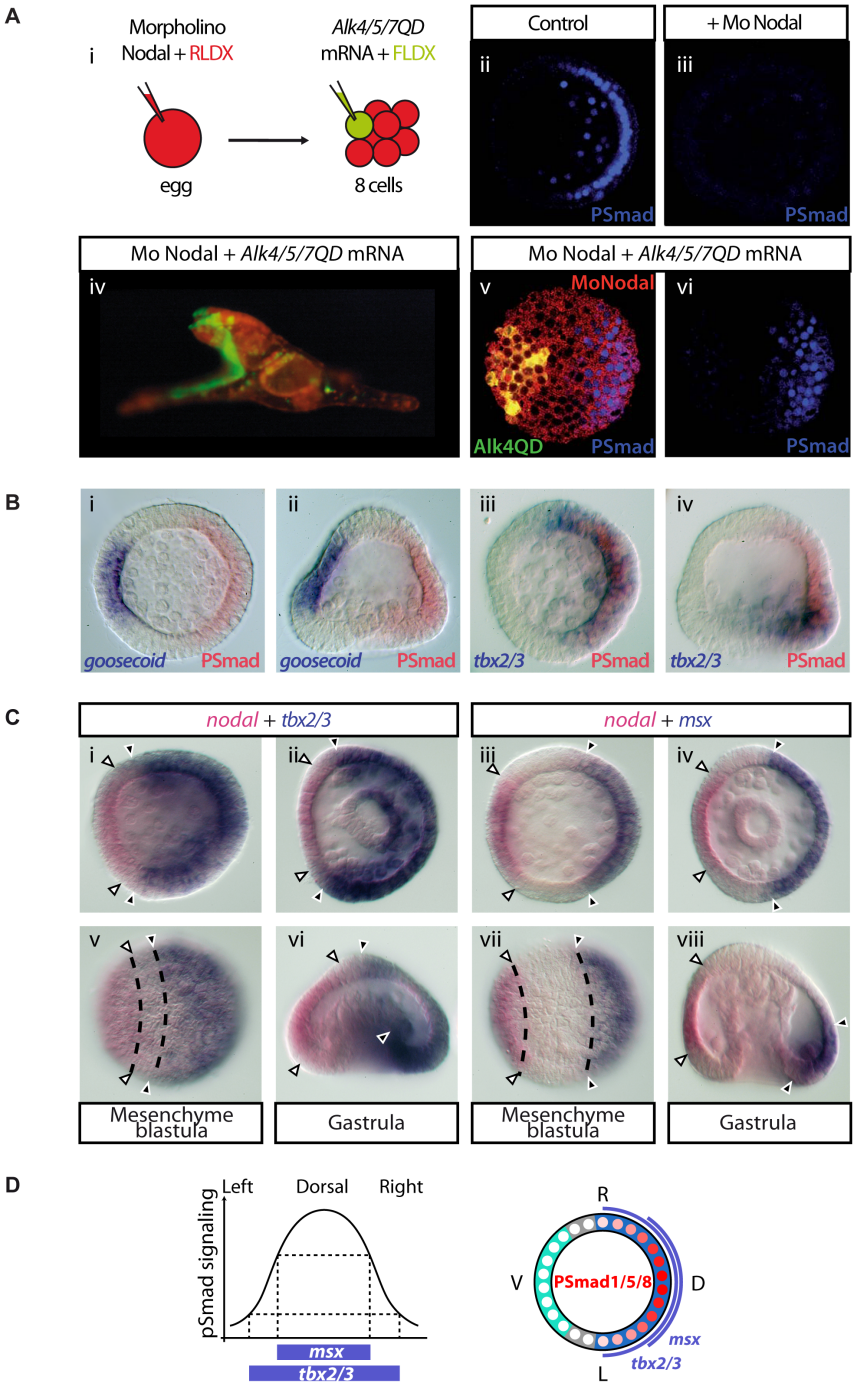
Ventral Nodal signals induce BMP2/4 translocation to the dorsal side where it activates *tbx2/3* and *msx* in nested patterns. (A) (i) Scheme of the experiment. pSmad1/5/8 immunostaining at the mesenchyme blastula stage in control embryo (ii), *nodal* morpholino injected embryo (iii), and embryos injected with *nodal* morpholino at the one-cell stage then injected into one blastomere with *alk4/5/7QD* mRNA at the eight-cell stage (v, vi). The *nodal* morpholino was injected together with a red fluorescent dextran and the *alk4/5/7QD* mRNA with a green fluorescent dextran. Both tracers can be seen in the fully rescued embryo in (iv). (B) Combined in situ hybridization and pSmad1/5/8 immunostaining reveal that BMP signaling is restricted to the dorsal side of the embryo. (i, ii) In situ hybridization with a probe for the ventral marker gene *goosecoid* (blue) confirms that *goosecoid* transcripts are expressed on the opposite side of the pSmad1/5/8 immunostaining (red). In contrast, *tbx2–3* transcripts (blue) are always found on the same side as the pSmad1/5/8 immunostaining signal (red) (iii, iv). (C) Nested expression of dorsal marker genes along the D/V axis. Double in situ hybridizations with *nodal* and *tbx2/3* (i, ii, v, vi) or *nodal* and *msx* (iii, iv, vii, viii) probes. *tbx2/3* is expressed in the whole dorsal ectoderm territory adjacent to the ciliary band. In contrast, *msx* [43] is expressed in a subdomain of the dorsal ectoderm more distant from the ciliary band. Expression of both *msx* and *tbx2/3* is regulated by BMP2/4 (Lepage et al. unpublished data). The embryos in (i–iv) are viewed from the animal pole. The embryos in (v–viii) are viewed from the side. (v) and (vii) are surface views. (D) Activation of target genes by the BMP2/4 morphogen gradient. *msx* and *tbx2/3* are expressed in nested patterns centered on the dorsal midline.

To test directly if Nodal activates BMP signaling within the ventral ectoderm or if it induces a BMP signal that acts as a relay to specify the dorsal side, we performed pSmad staining on *nodal* morpholino injected embryos rescued by ectopic expression of *alk4/5/7QD* into one blastomere at the eight-cell stage. As expected, the pSmad staining was abolished in *nodal* morpholino injected embryos (Figure 6Aiii), consistent with the absence of *BMP2/4* transcripts in the *nodal* morphants [16]. In contrast, pSmad staining was restored in all the embryos injected with the *nodal* morpholino and later rescued with the activated *alk4/5/7* mRNA (Figure 6Aiv–vi). Most strikingly, this pSmad staining was detected in a territory located not on the same but on the opposite side of the Alk4/5/7 expressing clone, the progeny of which was later restricted to the ventral side (Figure 6Aiv). Combined immunostaining with this antibody together with in situ hybridization with dorsal and ventral marker genes confirmed that in all cases, the pSmad nuclear staining was found on the opposite side of the territory expressing the ventral marker *goosecoid* (Figure 6Bi, ii) and on the same side as the territory expressing the dorsal marker *tbx2-3* (Figure 6Biii, iv). Interestingly, a number of dorsally expressed marker genes including *tbx2/3* and *msx* [42],[43] are expressed in nested partially overlapping patterns along the D/V axis (Figure 6C Lepage et al. unpublished data). The finding that pSmad1/5/8 staining is graded along the D/V axis and the nested expression of BMP2/4 target genes such as *tbx2/3* and *msx* both suggest that BMP2/4 may act as morphogen and may regulate different target genes as a function of its concentration along the D/V axis (Figure 6D).

Taken together, these results indicate that cells that receive a high level of BMP signal in the embryo are not located on the ventral side but on the dorsal side of the embryo. They also show that activation and translocation of pSmad1/5/8 into the nucleus in these dorsal cells is dependent on the Alk3/6 receptor and on the BMP2/4 ligand. They reveal that Nodal induces a BMP signal in the ventral ectoderm that activates BMP signaling in dorsal cells and therefore that in the sea urchin embryo, the territories where the signal is expressed and where the signal is received are on the two opposite sides of the embryo.

### The BMP Antagonist Chordin Is Co-Expressed with BMP2/4 in the Presumptive Ventral Region

**Figure 7:**
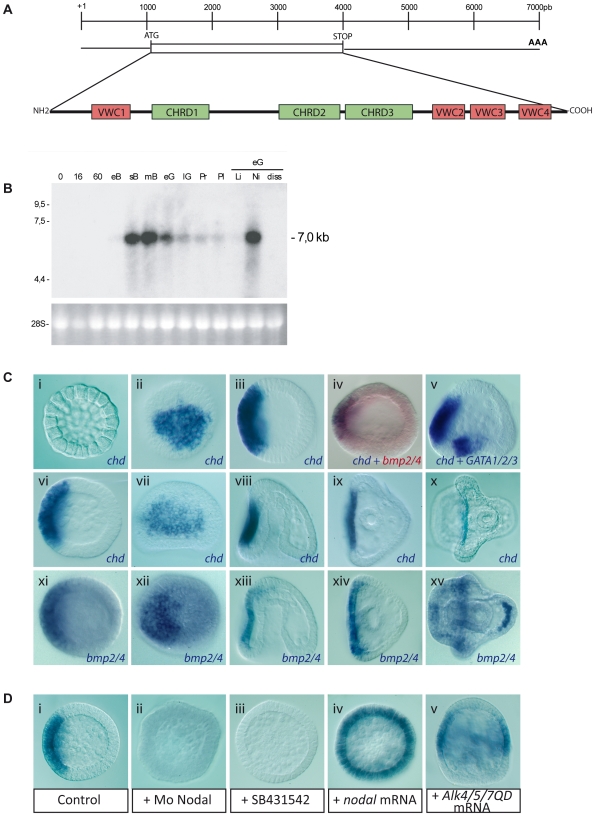
Sea urchin *chordin* is an early zygotic transcript expressed in the ventral ectoderm downstream of Nodal signaling. (A) Structure of the Chordin protein and cDNA. VWC, von Willebrand factor type C; CHRD, Chordin domain. (B) Northern blot analysis of *chordin* transcripts during sea urchin development. Embryonic stages are egg (O), 16 cells (16), 60 cells (60), early blastula (eB), swimming blastula (sB), mesenchyme blastula (mB), early gastrula (eG), late gastrula (lG), prism (Pr), and pluteus (Pl). (Li), embryos treated with lithium; (Ni), embryos treated with nickel; (Diss), dissociated embryos. Loading control is 28S mRNA. *chordin* is zygotically expressed starting at the swimming blastula stage. Its expression increases at the mesenchyme blastula stage, then decreases progressively during gastrulation up to the pluteus stage. *chordin* transcripts are not detected in dissociated or lithium treated embryos but are overexpressed in nickel treated embryos. (C) Spatial expression analysis of the *chordin* and *bmp2/4* transcripts by in situ hybridization. Embryonic stages are: 128 cell-stage (i), swimming blastula (ii–iv, xi, xii), mesenchyme blastula (v, vi), gastrula (vii, viii, xiii), late gastrula (ix, xiv), early pluteus (x, xv). (v) is a double in situ hybridization with *chordin* and *GATA1/2/3*, which is expressed in the non-skeletogenic precursors on the ventral side of the vegetal plate, while (iv) is a double in situ hybridization with *chordin* and *bmp2/4* probes showing that the two genes are expressed in a highly similar pattern. *chordin* expression begins at the swimming blastula stage and is strictly restricted to the ventral ectoderm up to the early pluteus stage where it is only maintained in a subdomain of the ciliary band. Note that at the pluteus stage, BMP2/4 expression in the ventral ectoderm has faded while strong expression is initiated in the dorsal-most skeletogenic mesenchyme cells (xv). (D) In situ hybridization of *chordin* transcripts at the mesenchyme blastula stage in a control embryo (i), or in an embryo injected with either the *nodal* morpholino (ii), *nodal* mRNA (iv), *alk4/5/7QD* mRNA (v), or treated with the Nodal receptor inhibitor SB431542 (iii).

The finding that BMP2/4 is produced on the ventral side but that BMP signaling is active on the dorsal side raised two intriguing questions: what prevents BMP2/4 from signaling in ventral cells and what promotes diffusion of BMP2/4 towards the dorsal side? In other systems, secreted BMP inhibitors such as Chordin in vertebrates or Sog in *Drosophila* are key regulators of the activity of BMP signals that sequester BMP ligands and prevent them from binding to and activating their receptors while allowing them to diffuse over long distances [1]. We thus isolated a *P.lividus* cDNA encoding Chordin and examined the expression of this gene during early development of the sea urchin embryo. Like the vertebrate Chordin and *Drosophila* Sog proteins, the sea urchin Chordin protein contains a hydrophobic leader sequence and four cysteine rich regions that are present in the same relative positions (Figure 7A and Figure S4). Northern blot analysis indicated that *chordin* is a strictly zygotic message that starts to accumulate in the embryo after hatching. Its expression peaks at the mesenchyme blastula stage, then decreases progressively during gastrulation and pluteus stages (Figure 7B). Chordin transcripts are downregulated in embryos treated with the vegetalizing agent lithium or in cultures of dissociated blastomeres but are overexpressed in embryos treated with the ventralizing agent nickel chloride (Figure 7B). Consistent with these observations, in situ hybridization revealed that during blastula and gastrula stages, the *chordin* (Figure 7Cii–x) and BMP2/4 genes (Figure 7Cxi–xiv) are transcribed in highly similar patterns within the ventral ectoderm, the BMP2/4 territory being slightly larger than the *chordin* expression domain at mesenchyme blastula and gastrula stages. Starting at the pluteus stage however, *BMP2/4* expression shifted to the dorsal skeletogenic mesenchymal cells while *chordin* remained expressed in a subdomain of the ciliary band (Figure 7Cx, xv). Like all the genes expressed in the ventral ectoderm of the sea urchin embryo identified so far, *chordin* is under the control of Nodal signaling: *chordin* transcripts are absent from embryos injected with *nodal* morpholinos and are ectopically expressed throughout the ectoderm following activation of the Nodal pathway (Figure 7D). We conclude that *chordin* and *BMP2/4* are expressed downstream of Nodal signaling. Thus, unlike in most organisms in which Chordin and BMPs are expressed in mutually exclusive regions, in the sea urchin embryo, their expression territories are congruent.

### Chordin Is Required for the Spatial Restriction of pSmad1/5/8 to Dorsal Cells

**Figure 8:**
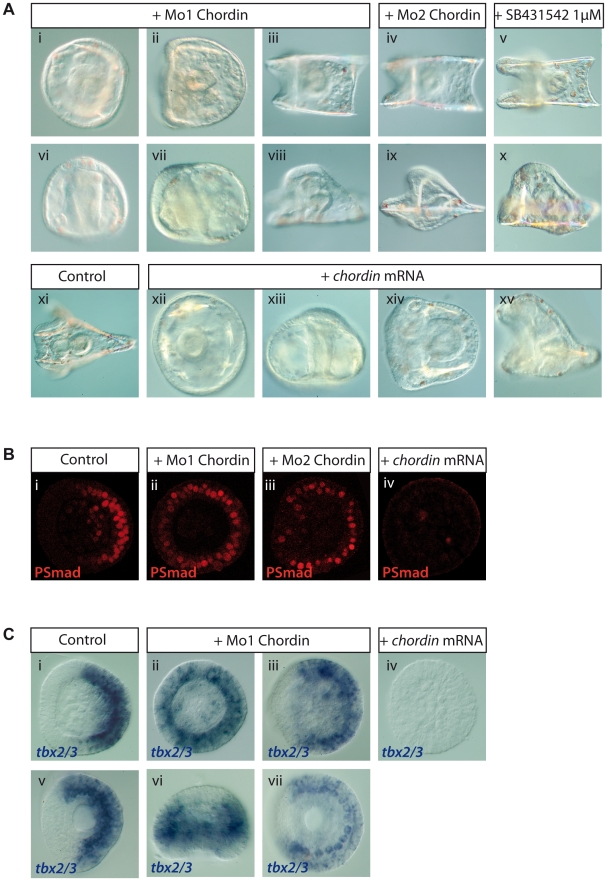
Chordin is required for patterning the D/V axis and for preventing pSmad1/5/8 signaling on the ventral side. (A) Blocking Chordin function using antisense morpholino oligonucleotides severely perturbs D/V polarity. About 80% of the embryos injected with Mo1 chordin at either 1 or 1.5 mM developed with a radialized phenotype with numerous ectopic spicules (i, vi). The remaining embryos were polarized along the D/V axis but failed to elongate and conserved a round shape (ii, vii). In some experiments, however, injection of the same doses of morpholino produced a milder but very reproducible phenotype characterized by a luge-like shape due to the parallel growth of the body rod spicules, and the presence of a proboscis in the animal region in place of the oral arms (iii, iv, viii, ix). Injection of Mo2 *chordin* at 0.6 mM produced predominantly the luge-like phenotype (iv, ix). The strong and mild phenotypes were observed in multiple experiments, one phenotype or the other prevailing in a given experiment depending on the batch of embryos. Treatments with low doses (1 μM) of the Alk4/5/7 inhibitor SB431542 phenocopy the mild *chordin* morpholino phenotype (v, x). In situ hybridization performed on these SB431542 treated embryos showed that expression of *chordin* is strongly reduced compared to controls (Lepage et al. unpublished data). Overexpression of *chordin* mRNA disrupted D/V patterning and caused complete (xii, xiii) or partial (xiv, xv) radialization. Here some variability was observed within a given experiment, with about half of the embryos developing with the strong phenotype and the other half with the milder phenotype (*n*>500; 5 experiments). Strongly affected embryos showed numerous ectopic spicules, a straight archenteron, and never elongated while less affected embryos acquired a bilateral symmetry but failed to elongate. (B) pSmad1/5/8 immunostaining of mesenchyme blastula stage embryos. While control embryos showed a restricted dorsal nuclearization of pSmad1/5/8 (i), embryos injected with the Mo1 *chordin* morpholino showed a broad expansion of the nuclear staining to the whole circumference of the ectoderm (ii). Embryos injected with Mo2 *chordin* also showed an expansion of the pSmad staining (iii), but it appeared less dramatic than that observed with Mo1 *chordin*. Conversely, pSmad1/5/8 staining was either not detectable or strongly reduced in most embryos (80%, *n* = 10) injected with the *chordin* mRNA (iv). (C) In situ hybridization showing *tbx2/3* transcripts expressed on the dorsal side in control embryos at mesenchyme blastula (i) and gastrula (v). The expression domain of *tbx2/3* was strongly expanded in most (30/40) embryos injected with a morpholino directed against *chordin* (ii, vi) covering the whole circumference of the ectoderm, while in the remaining embryos it appeared only slightly expanded (iii). This expansion of *tbx2/3* in *chordin* morpholino injected embryos is still visible at the gastrula stage but is less dramatic since most embryos at this stage show a ventral restriction of *tbx2/3* expression (vii). Injection of *chordin* mRNA causes the opposite effect and eliminates *tbx2/3* expression (*n* = 29) at the mesenchyme blastula stage (iv).

To test if Chordin is required to restrict BMP signaling to the dorsal side, we attempted to block its function with antisense morpholino oligonucleotides (Figure 8). Injection of Morpholinos oligonucleotides directed against the translation initiation codon of *chordin* (Mo1 *chordin*) produced a range of phenotypes of various severity. The most severely affected embryos were either completely radialized as shown by the presence of multiple ectopic spicules (Figure 8Ai, vi) or displayed profound defects in the establishment of D/V polarity such as the absence of ventral arms and dorsal apex but still retained a D/V polarity (Figure 8Aii, vii). The milder phenotypes were characterized by a poorly differentiated animal region that conserved a round shape and the absence or underdevelopment of the oral arms. A very peculiar and characteristic skeletal defect was observed in these embryos: the body rods spicules grew parallel instead of forming a triangle in the dorsal region, giving the embryos a luge-like shape (Figure 8Aiii, viii). These phenotypes (abnormal patterning of the ectoderm in the animal hemisphere and parallel growth of the spicules) were consistently observed following injection of a second non-overlapping morpholino directed against the 5′ UTR of the *chordin* transcript (Mo2 *chordin*) (Figure 8Aiv, ix). Interestingly this phenotype is also identical to the phenotype obtained following partial inhibition of Alk4/5/7 function using low doses of the SB431542 inhibitor, which strongly reduce *chordin* expression (Figure 8Av, x and unpublished data).

To test if Chordin is required to prevent BMP2/4 signaling on the ventral side, we monitored the distribution of pSmad1/5/8 in wild type embryos and in embryos injected with morpholino oligonucleotides against the *chordin* transcript (Figure 8B). In wild type embryos at mesenchyme blastula stage, the pSmad1/5/8 pattern was highly reproducible and staining was consistently detected in half of the embryo corresponding to the dorsal region. In embryos injected with either the morpholino directed against the translation start site or the morpholino directed against the 5′ UTR, however, a dramatic expansion of the pSmad1/5/8 pattern was observed such that staining was now detected both in dorsal and in ventral cells (Figure 8Bii, iii). Consistent with the expansion of the pSmad1/5/8 staining, these embryos showed an expansion of *tbx2/3* expression at mesenchyme blastula stages (Figure 8Cii, iii, vi). This expansion of *tbx2/3* expression was also visible at gastrula stages, although it appeared less dramatic at this stage, suggesting the existence of compensatory regulatory mechanisms (Figure 8Bvii). This result indicates that one function of Chordin is to prevent BMP2/4 from binding to and activating its receptor in the ventral ectoderm. Consistent with this idea, we found that the sea urchin Chordin protein has a very potent dorsalizing activity when overexpressed in zebrafish embryos mimicking the *swirl* mutant phenotype caused by inactivation of *bmp2* (Figure S5) [44]. To further test if Chordin contributes to the establishment of the pSmad1/5/8 pattern, we overexpressed it by mRNA injection into the egg. Overexpression of *chordin* strongly affected D/V polarity (Figure 8Axi–xv). In 30% of the embryos it inhibited development of the dorsal region and caused a strong radialization as indicated by the formation of ectopic spicules throughout the circumference of the embryo (Figure 8Axii, xiii). The remaining embryos developed with a bilateral symmetry, but development of the ventral arms and dorsal apex of the larva were reduced (Figure 8Axiv, xv). pSmad1/5/8 staining (Figure 8Biv) and *tbx2/3* expression (Figure 8Civ) was eliminated in these embryos.

Taken together these results demonstrate that in the sea urchin embryo as in other bilaterian models, Chordin plays a key role in secondary axis specification through the spatial restriction of BMP2/4 signaling.

### BMP2/4 Is Widely Diffusible, Even in the Absence of Chordin

That the cells expressing the BMP2/4 ligand are located on the ventral side while the cells receiving the signal are located on the dorsal side strongly implied that secreted BMP2/4 ligand could act over a long distance.

**Figure 9:**
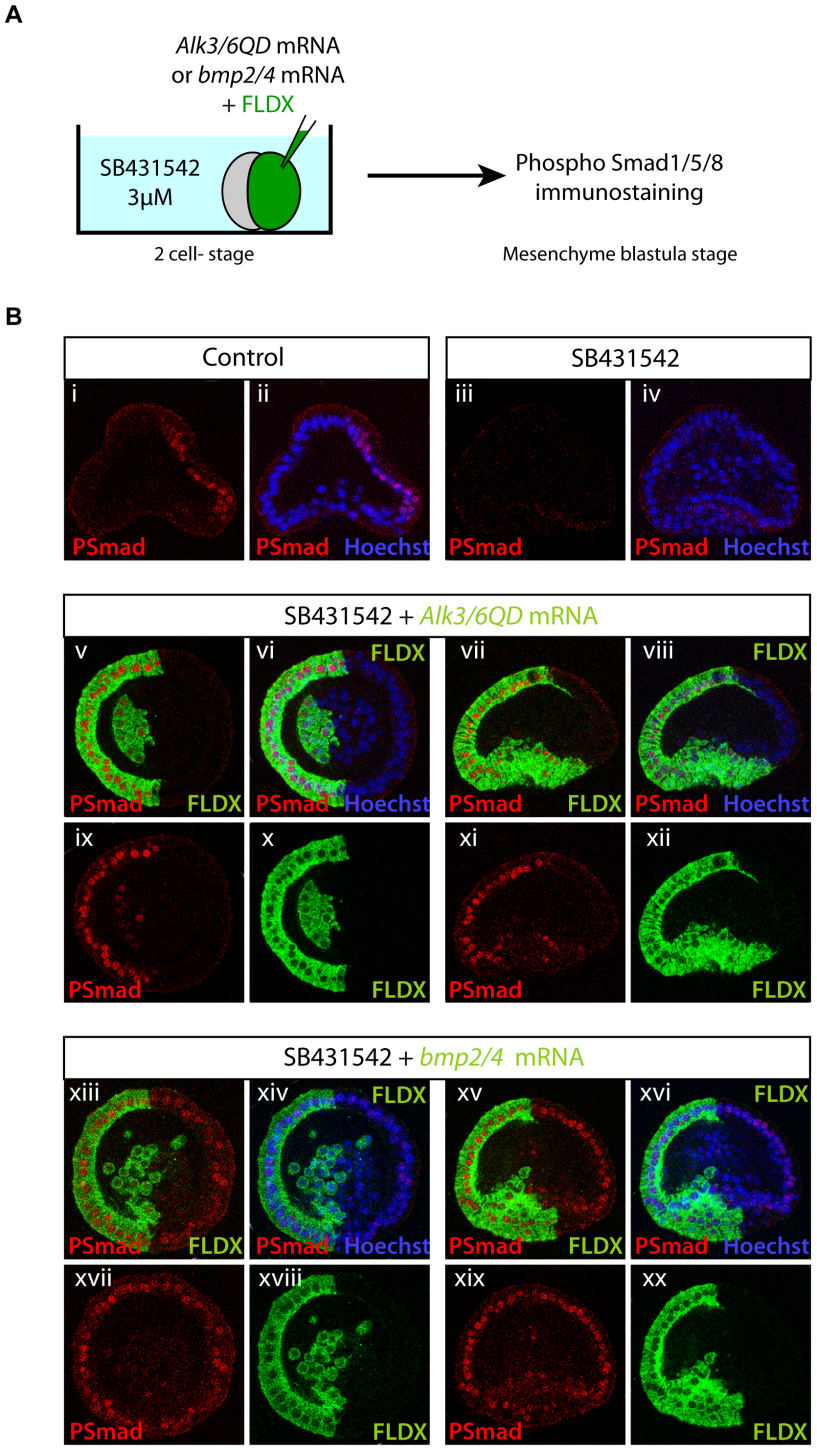
BMP2/4 is widely diffusible in the absence of Chordin. (A) Scheme of the experiment. At the two-cell stage, embryos were injected into one blastomere with either mRNA encoding the activated Alk3/6 receptor, *alk3/6QD*, or with the *bmp2/4* mRNA together with a green fluorescent dextran (FLDX) as a lineage tracer. To prevent *chordin* expression the embryos were treated continuously with the Nodal receptor inhibitor SB431542 starting soon after fertilization and up to the mesenchyme blastula stage when they were fixed and immunostained with the anti-pSmad1/5/8 antibody. (B) (i, ii) Control embryos showing asymmetrical nuclear pSmad1/5/8 staining in the dorsal half of the embryo. Embryos treated with SB431542 did not show any pSmad1/5/8 signal in any cell (iii, iv). Embryos injected at the two-cell stage with mRNA encoding Alk3/6QD, the activated BMP receptor, and treated with SB431542 displayed strong nuclear staining in half of the embryo. This staining strictly co-localized with cells that received the *alk3/6QD* mRNA (v, ix). Embryos injected at the two-cell stage with mRNA encoding BMP2/4 and treated with SB431542 showed nuclear pSmad1/5/8 staining in all cells of the ectoderm (xiii–xx). Note that the territory showing nuclear pSmad includes as many cells that received the *bmp2/4* mRNA as cells that did not receive the mRNA (xiii, xv). The pSmad signal is indicated by the red fluorescence (i, iii, ix, xi, xvii, xix), position of the nuclei is revealed by Hoechst staining (ii, iv, vi, viii, xiv, xvi), and the lineage tracer is detected by the green fluorescence of the fluoresceinated dextran (x, xii, xviii, xx). Sagittal confocal optical sections of representative embryos (i–iv, vii, viii, xi, xii, xv, xvi, xix, xx). Latitudinal confocal optical section of representative embryos (v, vi, ix, x, xiii, xiv, xvii, xviii).

To test this possibility and to investigate the role of Chordin in BMP2/4 diffusion, we injected mRNA encoding either Alk3/6QD, the activated BMP receptor, or the secreted BMP2/4 ligand into one blastomere at the two-cell stage. Then at the mesenchyme blastula stage, embryos were fixed and immunostained with the anti-phospho-Smad1/5/8 antibody (Figure 9A). To prevent synthesis of Chordin and distinguish the ectopic from the endogenous BMP2/4 signal, the experiment was performed in the presence of the Alk4/5/7 inhibitor SB431542 added after fertilization. Embryos treated with SB431542 fail to express Nodal target genes such as *chordin* (Figure 7Diii) and *BMP2/4* (unpublished data) and therefore the endogenous nuclear pSmad1/5/8 staining should be eliminated [14],[16]. As predicted, the dorsal phospho-Smad1/5/8 staining present in control embryos at blastula stages was absent in SB431542 treated embryos (Figure 9Bi–iv). Embryos injected with mRNA encoding the activated form of the receptor Alk3/6QD at the two-cell stage and treated with the Alk4/5/7 inhibitor showed a strong nuclearization of pSmad1/5/8, but this signal was restricted to the clone of cells that received the mRNA (Figure 9Bv–xii). In contrast, injection of *BMP2/4* mRNA at the two-cell stage combined with SB431542 treatment resulted in a dramatic increase of nuclear pSmad1/5/8 staining, all cells of the ectoderm displaying strong nuclear staining (Figure 9Bxiii–xx). This indicates that in a SB431542 context and therefore even in the absence of the carrier protein Chordin, BMP2/4 is widely diffusible and can trigger long-range signaling within the ectoderm.

### Transcription of the Heparin Sulphated Proteoglycan *Glypican 5* Is Regulated by BMP2/4 Signaling

**Figure 10:**
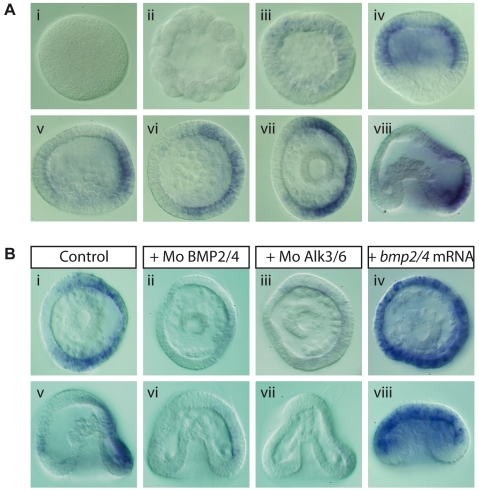
Glypican 5 expression is progressively restricted to dorsal cells and is regulated by BMP2/4-Alk3/6 signaling. (A) Expression pattern of *glypican 5* during embryogenesis. (i) egg; (ii) 60-cell stage; (iii) early blastula; (iv) swimming blastula; (v, vi) mesenchyme blastula; (vii, viii) gastrula. (B) After an initial phase of expression in a belt of cells around the equator (iv), *glypican 5* expression becomes strongly asymmetric (v–viii). (B) The dorsal expression of *glypican 5* was abolished in *bmp2/4* (ii, vi) or *alk3/6* (iii, vii) morphants. In contrast, *glypican 5* was ectopically expressed throughout the ectoderm in *bmp2/4* (iv, viii) or *alk3/6QD* (unpublished data) overexpressing embryos.

Proteoglycans such as Glypicans have emerged as major regulators of morphogen stability and mobility across tissues [45]–[47]. We therefore examined the expression of the genes encoding proteoglycans of the Glypican family, which have been shown to play crucial roles in movement of BMPs across a field of cells. The sea urchin genome contains two *glypican* genes, *glypican 5* and *glypican 6* [29],[48]. *glypican 6* is abundantly expressed maternally, and its transcripts are uniformly distributed during cleavage, blastula, and gastrula stages (unpublished data). In contrast, transcription of *glypican 5* is first activated at blastula stages in a belt of cell that includes the whole presumptive ectoderm except the animal pole region (Figure 10Aiv). Interestingly, starting at mesenchyme blastula stage, *glypican 5* expression becomes restricted to the dorsal ectoderm (Figure 10Av–viii). This suggested that *glypican 5* expression at this stage may be regulated by BMP2/4 signaling. Indeed, blocking BMP2/4 or Alk3/6 function eliminated expression of *glypican 5* at gastrula stages (Figure 10Bii, iii, vi, vii). Conversely, ectopic expression of BMP2/4 or Alk3/6QD (unpublished data) induced strong expression of *glypican 5* throughout the ectoderm (Figure 10Biv, viii). This indicates that transcription of this regulator of BMP signaling is itself under the control of BMP signaling and may thus be involved in a positive feedback loop.

## Discussion

**Figure 11:**
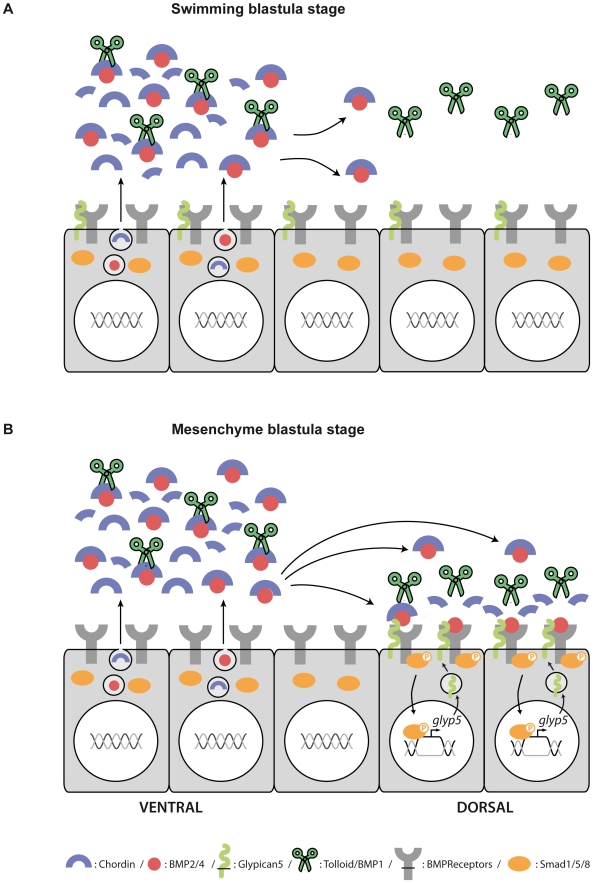
A model for morphogen gradient formation in the sea urchin embryo. (A) *chordin* and *bmp2/4* are both expressed in the ventral ectoderm downstream of Nodal while the presumed Chordin protease Tolloid/BMP1 [83], the BMP2/4 receptor *alk3/6*, and *glypican 5* are expressed widely throughout the ectoderm at blastula stages. BMP2/4 ligands expressed on the ventral side of the embryo are immediately complexed with Chordin and cannot bind to their receptors. Therefore, on the ventral side of the embryo, no free BMP2/4 ligand can bind to BMP receptors since an excess of Chordin is able to constantly reform Chordin/BMP2/4 complexes. (B) The Chordin/BMP2/4 complexes produced on the ventral side of the embryo can nevertheless diffuse towards the dorsal side of the embryo where Tolloid/BMP1 releases free BMP2/4 ligands that, in the absence of Chordin, can bind to their receptors and trigger Smad1/5/8 phosphorylation and nuclearization. BMP signaling on the dorsal side up-regulates expression of *glypican 5*, which may facilitate BMP2/4 mobility and BMP2/4 binding to its receptor, thereby reinforcing BMP2/4 signaling via a positive feedback loop.

Sea urchin Nodal has a remarkable ability to organize the embryonic D/V axis, and understanding the underlying molecular mechanism should provide important insights into the evolution of metazoan axial patterning. Based on the ability of ectopic *nodal* mRNA to rescue patterning over the whole D/V axis in rescue experiments, we had speculated that Nodal most likely required a relay molecule to induce the dorsal ectoderm [16]. However, direct evidence for the existence of a relay was lacking. The identity of the relay molecule(s) acting as the dorsal inducer(s) had not been demonstrated and direct evidence that BMP signaling was active on the dorsal side was missing. Similarly, the function of conserved regulators of BMP signaling such as Chordin in patterning the D/V axis was not known. In this study we have addressed these issues. We performed a functional analysis of several key genes involved in D/V patterning in the sea urchin embryo: the BMP ligand *BMP2/4*, the BMP receptor *alk3/6*, the BMP antagonist *chordin*, and the extracellular regulator *glypican5.* By using the rescue experiment as a functional assay, we provided a conclusive demonstration that Nodal does not work as a morphogen but requires a relay identified as BMP2/4. By using anti-phospho-Smad immunostaining as a direct read-out of BMP signaling, we discovered that BMP2/4 produced in ventral cells activates BMP signaling on the dorsal side of the embryo, and therefore that in the sea urchin embryo, the dorsal ectoderm is induced by ventrally produced signals. We also found that in the sea urchin, *chordin* is co-expressed with *BMP2/4* but that despite this very unusual expression pattern, it has a conserved role in antagonizing BMP2/4. These results allow us to provide a new model for patterning along the D/V axis of the sea urchin embryo (Figure 11). They also show that although genes used for D/V patterning such as *BMP2/4* and *chordin* are conserved in this organism, the relationships between their expression patterns are dramatically different from what was described so far in other models. These findings provide valuable information to understand the evolution of the pathways used for D/V axis specification in the ancestor of deuterostomes. They also raise important and intriguing new questions regarding the regulation of BMP activity and the regulation of BMP diffusion in embryos.

### Nodal Acts via a Relay to Induce Dorsal Cell Fates

In zebrafish, ligands of the Activin/Nodal family have been proposed to control antero-posterior patterning and to work as long-range morphogens [49]. In particular, Squint has been demonstrated to work as a morphogen capable of diffusing over several cell diameters to induce target genes [19],[20]. In sea urchins, however, the ability of *nodal* mRNA to efficiently rescue normal development and to generate a morphologically normal pluteus larva is not very sensitive to the concentration of *nodal* mRNA injected, arguing against the morphogen hypothesis. Furthermore, it has been shown recently in vertebrates that Nodal antagonists such as Lefty spatially restrict the activity of Nodal ligands. Lefty factors are induced by Nodal signaling but are thought to diffuse faster than Nodal outside their expression territory, inhibiting the ability of Nodal to auto-activate and to induce downstream target genes. In the sea urchin embryo, Nodal and Lefty most likely obey the same rules [18] and constitute a typical reaction diffusion system. Further evidence for a lack of long-range action of Nodal is the demonstration of its short range of action in the animal pole region [26].

Nodal does not need to diffuse over a long range to rescue the dorsal structures, and therefore it must work by inducing a relay. We demonstrated that by comparing the effects of ectopically expressing the secreted Nodal ligand to the effects of ectopically activating the receptor complex that transduces Nodal signals in early embryos previously injected with a *nodal* morpholino. Furthermore we showed that in this rescue experiment, the Nodal receptor complex not only specifies ventral cell fates in a cell autonomous manner, but that it causes the BMP pathway to be activated on the opposite side demonstrating that Nodal does not work as a morphogen but that it induces a long range relay molecule that regulates morphogenesis of the dorsal region. It therefore follows that specification and patterning of the D/V axis of the sea urchin embryo does not result from the interactions between a BMP expressing ventral centre and a Chordin expressing dorsal centre, as in vertebrates, but that the activities of these two centres, which are both induced by Nodal, are concentrated within the ventral ectoderm.

### The BMP Pathway Is Activated on the Dorsal Side of the Embryo by a Ventrally Produced BMP2/4 Ligand

In all the organisms where D/V patterning by BMP signaling has been studied, BMPs always act within the territory in which they are expressed. In this study, however, we provided several lines of evidence that, in the sea urchin embryo, specification of dorsal cell fates stringently relies on expression of the BMP2/4 ligand on the ventral side, but with the BMP molecule being translocated to the dorsal side where it activates BMP signaling. First, we showed that BMP2/4 and the type I receptor Alk3/6 are not required for specification of ventral cell fates but are essential for specification of the dorsal region of the embryos. In the absence of BMP2/4 or Alk3/6 function, the expression of ventral marker genes is unaffected, the mouth opens, but the expression of dorsal marker genes is abolished and the dorsal region of the embryo is not specified. Second, by directly visualizing the activation of the BMP pathway at blastula stages using pSmad1/5/8 immunostaining, we showed that cells that receive a high level of BMP signals are located on the dorsal side of the embryo, opposite to the side where the Nodal pathway is active and where BMP2/4 is expressed. Third, we showed that BMP signaling monitored by this anti p-Smad antibody is critically dependent on BMP2/4 and Alk3/6 function. Finally, we showed that BMP2/4 can signal over a long range from one side of the embryo to the opposite side. This does not rule out the possibility that other BMP ligands may be important for D/V patterning in the sea urchin embryo as shown in the case of Dpp and Scw in *Drosophila*. However, the abolition of pSmad1/5/8 staining in dorsal cells following inhibition of BMP2/4 function indicates that BMP2/4 is critically required for activation of Smad1/5/8 during gastrulation and that in its absence, other potential BMP ligands are not sufficient to activate BMP signaling in these dorsal cells. Intriguingly, down regulation of BMP2/4 abolished the pSmad1/5/8 staining while down regulation of Alk3/6 drastically reduced but did not eliminate pSmad1/5/8 staining. The residual pSmad1/5/8 staining observed in the *alk3/6* morpholino injected embryos may be due to maternal Alk3/6 protein or it may reflect the activity of Alk1/2, the second BMP type I receptor present in the sea urchin genome, which could partially compensate for the loss of Alk3/6. However, the phenotype of *alk3/6* morphants and the absence of expression of dorsal ectodermal markers in these embryos indicate that this residual nuclear phospho-Smad1/5/8 is not sufficient to trigger the genetic networks leading to specification of dorsal cell fates.

### D/V Patterning of the Sea Urchin Embryo: An Extreme Case of BMP Translocation

The mechanisms of BMP gradient formation have been best studied in *Drosophila* (reviewed in [1]). The prevalent view is that *Drosophila* Sog binds to Dpp and inhibits Dpp signaling within and near its territory of expression but that it also prevents receptor mediated internalization and turnover and thereby allows translocation of Dpp towards the dorsal midline where it is released by the metalloprotease Tolloid. Both genetic and biochemical studies in *Drosophila* have provided evidence for this translocation mechanism as a way to concentrate Dpp in a subdomain of a territory in which it is expressed uniformly. A similar situation is found in vertebrates, with *BMP2/4* and *chordin* being expressed in mutually exclusive territories in the ventral and dorsal regions. Furthermore, experimental manipulations in *Xenopus* indicate that a shuttling mechanism of BMP ligands is responsible for translocation of BMP ligands ventrally through association with Chordin [50].

In the sea urchin embryo, BMP2/4 signaling is activated in a domain located outside its domain of expression, on the opposite side of the embryo. In this respect, it provides one of the most extreme examples known so far of translocation of a BMP ligand. It will be interesting in the future to dissect the mechanism and to identify the proteins involved in this process. Additional extracellular proteins are likely involved in the regulation of BMP2/4 translocation and activity, including the metalloprotease Tolloid, and the adaptator protein Twisted gastrulation, both of which have been shown to participate in the shuttling of BMP ligands in vertebrates and *Drosophila* [1]. The sea urchin genome contains a family of more than 10 Tolloid/BMP1 related genes, the functions of which are poorly understood [51]–[53].

### BMP2/4 as the D/V Morphogen in the Sea Urchin Embryo

Our finding that there is a gradient of nuclear pSmad1/5/8 along the D/V axis together with the nested patterns of expression of *tbx2/3* and *msx*, two BMP target genes, strongly suggests that BMP2/4 is acting as a morphogen along the D/V axis. One would therefore predict that *msx*, which is expressed in the domain of highest BMP signaling, may be activated by high levels of BMP signaling while *tbx2/3*, which is expressed in the whole dorsal ectoderm, may require lower levels of BMP signals. The next question to answer will be to determine whether the graded pSmad1/5/8 pattern reflects a gradient of BMP2/4 protein or a gradient of BMP2/4 activity. One hypothesis is that BMP2/4 may be transported toward the dorsal side to form a D/V gradient of protein. An alternative hypothesis may be that BMP2/4 is present at homogenous levels throughout the dorsal side but that one of its antagonist is present in an opposite ventral-dorsal gradient. It will therefore be important to visualize the distribution of BMP2/4 protein in the extracellular space and to monitor BMP2/4 shuttling and accumulation on the dorsal side as described in *Drosophila* and vertebrates [54],[55].

### Chordin Prevents BMP Signaling on the Ventral Side

Our finding that pSmad1/5/8 signaling is restricted to dorsal cells while the source of BMP2/4 ligand is located on the ventral side raised an intriguing question: What prevents BMP2/4 from activating its receptor in the ventral ectoderm? The distribution of the BMP2/4 receptors cannot explain this spatial restriction since Alk3/6 is expressed ubiquitously. Our finding that the *chordin* gene is expressed abundantly in the ventral ectoderm and that its product acts as an inhibitor of BMP2/4 signaling resolves this issue. The finding that *chordin* expression is regulated by Nodal signaling also helps to understand how a genetic circuit leading to specification of the dorsal ectoderm can be embedded into the genetic program leading to specification of the ventral ectoderm: Nodal induces BMP2/4 in the ventral ectoderm, and at the same time, it induces a potent antagonist of BMP signaling that prevents signaling in the ventral ectoderm and promotes diffusion of BMP2/4 outside this territory.

In *Drosophila sog* mutants, extracellular Dpp binds to all cells in the dorsal domain leading to broad pSmad staining. This is similar to the situation in the sea urchin embryo. In *chordin* morphants, BMP signaling invades the ventral side of the embryo leading to ectopic expression of dorsal marker genes such as *tbx2/3*. Furthermore, overexpression of *chordin* eliminated pSmad1/5/8 signaling and suppressed expression of dorsal markers genes. Our results therefore establish that in the sea urchin embryo, like in all the other organisms where the function of this gene has been studied, *chordin* plays a key role in D/V patterning through the spatial regulation of BMP2/4 signaling. These results contradict the conclusions of a recent study performed in a different sea urchin species on the role of Chordin in sea urchin development [56]. Bradham et al. reported that both overexpression of *chordin* and injection of the *chordin* morpholino strongly perturbed D/V polarity and suppressed formation of the dorsal side and oral arms, but surprisingly these perturbations did not affect *tbx2/3* expression at late gastrula stage. The reasons for this discrepancy are not clear but we feel that explanations may reside in the different doses of RNA used, the different methods used for phenotypic characterization, and the different stages analyzed. In our study, we used doses of synthetic *chordin* mRNA up to 1 mg/ml while Bradham et al. used RNA concentrations one order of magnitude lower. Also, we performed both anti-phospho Smad1/5/8 staining and in situ hybridization at blastula stages while Bradham analyzed late gastrula embryos by in situ hybridization. At this late gastrula stage, the effects of the *chordin* morpholino on *tbx2/3* expression are much less apparent probably due to compensatory mechanisms (see Figure 8Cvii).

### BMP2/4 Is Widely Diffusible in the Absence of Chordin

The ability of BMP ligands to diffuse over long distances in the absence of carrier proteins such as Sog/Chordin is still a controversial issue. Early studies of *Drosophila* Sog mutants revealed that Dpp is widely diffusible in the presence of Sog but tightly localized in its absence, suggesting that diffusion of Dpp is spatially restricted [57]. The restricted diffusion of BMP ligands in the absence of carrier proteins such as Sog/Chordin has been proposed to be a key parameter and a condition for robustness in one of the mathematical models proposed to explain formation of the Dpp gradient. Limited ability of BMPs to diffuse in the absence of carrier proteins such as Chordin has also been documented in *Xenopus* [50]. In contrast, by examining embryos in which Dpp was ectopically expressed, Mizutani et al. found that the range of Dpp action was reduced but still substantial, consistent with free diffusion [58].

In this study, we examined the range of BMP2/4 signaling in embryos treated with the Nodal receptor inhibitor SB431542 that prevents *chordin* expression. We found that BMP2/4 is highly diffusible in these conditions. Therefore, our data argue in favor of free diffusion of BMP ligands in early embryos.

An interesting finding of our study that may explain the high diffusibility of BMP2/4 in the sea urchin embryo is that *glypican 5*, a cell-surface BMP binding protein, is expressed early in the dorsal and ventral ectoderm. Glypicans have been implicated as major regulators of BMP stability and/or BMP movement across fields of cells [45],[46],[59],[60]. In *Drosophila*, analysis of clones of cells deficient for or overexpressing Dally revealed that Dally regulates Dpp mobility, leading to the proposal that it is required to transmit the Dpp protein from cell to cell [45]. Interestingly, it has been shown that Dally promotes Dpp signaling and Dpp mobility in a non-autonomous manner [46]. For example, overexpressing Dally in the posterior compartment of the haltere, where it is normally absent, leads to a shift in Dpp signaling from the anterior to the posterior compartment [46],[61],[62]. On the basis of this and other observations, it was suggested that Dally biases Dpp mobility towards cells with higher level of Dally or, in other words, that cells with a high level of Dally attract Dpp [46]. Although asymmetries in *glypican* expression have been reported in the wing and haltere discs, differential *glypican* expression has never been reported to our knowledge in early embryos. Our finding of a strong asymmetry of *glypican 5* expression in the early sea urchin embryo, with high level in the dorsal region, distant from the source of BMP2/4 signals, strongly suggests that a similar mechanism may also be used in early embryos to shape the BMP morphogen gradient that is responsible for patterning the D/V axis.

In the *Drosophila* embryo, localized injection of mRNA encoding an activated form of Thickveins promotes accumulation of extracellular Dpp, implying the existence of a positive feedback loop [54]. This observation led to the hypothesis that BMP signaling induces synthesis of a hypothetical cell-surface BMP binding protein that could either reduce the interaction of ligands with an inhibitory component or enhance future ligand receptor interactions. Mathematical modeling of BMP signaling during D/V patterning as well as experimental studies in other signaling pathways predict that positive feedback circuits can convert graded inputs into all or no outputs leading to production of bistable signaling states in which there is a sharp transition between cells displaying a high level of signaling and cells with very low signaling [63],[64]. We speculate that the BMP2/4-*glypican 5* positive regulatory input we have characterized likely plays a role in intensifying BMP2/4-Alk3/6 interactions in the dorsal ectoderm.

### A New Model of D/V Axis Patterning in Echinoderms

Our results allow us to propose a new model of D/V patterning by BMP signals in the sea urchin embryo (Figure 11). Nodal signaling induces *BMP2/4* and *chordin* expression in the ventral ectoderm. High levels of Chordin on the ventral side prevent BMP2/4 from binding to its receptor. Therefore, BMP2/4-Chordin complexes diffuse or are transported toward the dorsal side resulting in formation of a shallow D/V gradient of BMP2/4 signaling. Glypican 5, which is expressed in the whole ectoderm at this stage, may also participate in this initial phase of BMP translocation. Starting at the mesenchyme blastula stage, BMP signaling then feeds back onto *glypican 5* expression. Based on the established activities of Glypicans as positive modulators of BMP signaling and BMP mobility in other systems, we speculate that this preferential expression of *glypican 5* may bias BMP2/4 mobility towards the dorsal side and may increase the ability of BMP2/4 to bind to its receptor. This may further reinforce the accumulation of BMP2/4 in dorsal cells and augment BMP signaling in dorsal versus ventral cells.

### BMP-Chordin and the Evolution of Neural Patterning

A conserved feature between *Drosophila* and vertebrates is that both Chordin and Sog display a proneural activity, via inhibition of BMP2/4 or Dpp function in the neurectoderm. Surprisingly, in embryos of the hemichordate *Saccoglossus kowalevkii*, which have a diffuse neural system, the BMP signaling pathway does not repress neural gene expression [3]. Echinoderms, like hemichordates, develop a rather diffuse nervous system. However, a notable difference between the nervous system of echinoderms and hemichordates embryos is that echinoderms have retained a strong polarization of their nervous system along the D/V axis, most neurons arising in the animal pole and in the ciliary band territory, which lies at the interface between the ventral and the dorsal ectoderm. In this study, we showed that inhibiting the function of BMP2/4 or its receptor Alk3/6 dramatically expanded the neurogenic territory of the ciliary band towards the dorsal side. This strongly suggests that, unlike in hemichordates, neural tissue formation in echinoderms requires inhibition of BMP signaling. Taken together, our results are consistent with a model for specification of the ectoderm in the sea urchin embryo that relies on the hypothesis that the default state of most of the ectoderm in the absence of Nodal signaling is a proneural, ciliary band-like fate and that Nodal and BMP2/4 act in cascade to specify, respectively, the ventral and the dorsal ectoderm, restricting the ciliary band to the territory located in between these two specified territories.

### BMP-Chordin and the Evolution of D/V Patterning

Based on the conservation of the molecular pathway used both to develop a secondary axis and to form a nervous system in *Drosophila* and vertebrates, it was proposed that this molecular pathway was already used for specification of the D/V axis and nervous system in their common bilaterian ancestor but that an inversion of the D/V axis had occurred during evolution [65]–[67]. According to this hypothesis, the common ancestor would have possessed a centralized nervous system and a plausible mechanism to explain the D/V axis inversion is that the mouth would have moved from one side of the embryo to the other or, that the trunk would have rotated 180° relative to the head [6]. A recent study showed that in Cephalochordates, the most basal chordates according to recent phylogenetic analyses, the expression domain of *chordin* and BMP2/4 retained the same orientation as in protostomes (i.e., BMP2/4 being dorsal and *chordin* ventral) [4], suggesting that any inversion occurred after the emergence of chordates or, alternatively, that a second inversion would have occurred independently in this lineage. In the hemichordate *Saccoglossus kowalevkii*, an “invertebrate like” BMP-chordin axis is also present, with *chordin* being expressed on the ventral side and BMP on the dorsal side [3].

**Figure 12:**
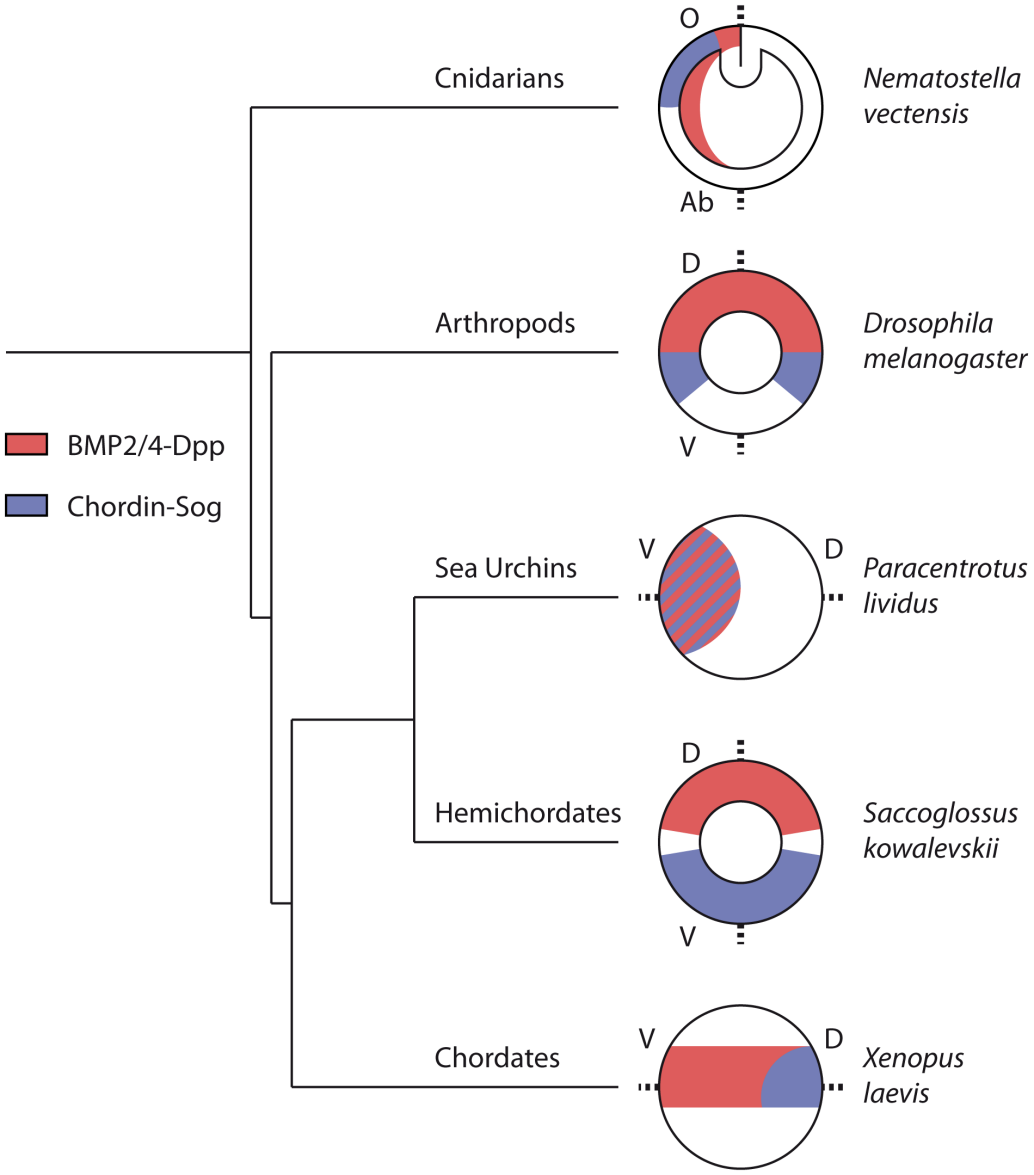
Schematic diagram comparing the expression domains of BMP2/4 and Chordin homologues in different experimental models. The relative expression patterns of BMP and *chordin* in organisms belonging to the main clades of metazoan (protostomes and deuteurostomes together with diploblastic cnidarians as outgroup) are depicted. O, oral; Ab, aboral; D, dorsal; V, ventral. While in most organisms *chordin* and BMP are expressed in mutually exclusive patterns, in the sea urchin and in cnidarians these genes are co-expressed on the same side of the embryo.

One novel finding of this study is that BMP2/4 and *chordin* are co-expressed in the ventral ectoderm of the embryo. The co-expression of BMP2/4 and *chordin* in the ventral ectoderm of the sea urchin embryo contrasts with the situation present in embryos of all the other bilaterians organisms (Figure 12). Thus, it indicates that although a BMP Chordin axis is present in the sea urchin embryo, this axis is defined by the activities of these factors and not by their expression territory.

Recent studies have shown that the *dpp* and *chordin* genes are present in the genomes of cnidarians, which are positioned phylogenetically as an outgroup to the bilaterians. Unexpectedly, in *Nematostella vectensis* gastrulae, the homologs of both genes are expressed asymmetrically along a secondary axis perpendicular to the oral-aboral axis. Even more intriguing is the fact that, in *Nematostella* like in sea urchin, *BMP2/4-dpp* and *chordin* are expressed on the same side of the embryo [68]–[71]. It has been suggested that animals with a radial symmetry could have developed a secondary axis that was lost later during evolution [72]. The asymmetrical expression of *dpp* and *chordin* in the *Nematostella* embryo could therefore reflect the presence of a cryptic D/V axis or a convergent use of BMP signaling for secondary axis specification. That *chordin* and *BMP2/4*-*dpp* are expressed in the same territory both in embryos of a basal deuterostome and in embryos of *N. Vectensis* raises the intriguing possibility that co-expression of BMP ligands and BMP antagonists such as Chordin on the same side of the embryo could have been the ancestral way to develop a secondary axis and the switch to opposite expression territories for the ligand and the antagonist a more recent evolutionary innovation. Another hypothesis is that in the two radially symmetrical organisms, the sea urchin and the sea anemone, BMP signaling may have evolved independently on the basis of co-expression. Functional analysis of the cnidarian *chordin* and *dpp* genes and investigation of the role of the BMP chordin signaling network in additional protostomes organisms are required to address these issues.

## Materials and Methods

### Animals, Embryos, and Treatments

Adult sea urchins (*Paracentrotus lividus*) were collected in the bay of Villefranche-sur-Mer. Embryos were cultured at 18°C as described previously [73],[74]. Fertilization envelopes were removed by adding 2 mM 3-amino-1,2,4 triazole 1 min before insemination to prevent hardening of this envelope followed by filtration through a 75 µm nylon net [16]. Treatments with SB431542 were performed by adding the chemical at 1, 3, or 5 µM diluted from stocks in DMSO, in 24 well plates protected from light. Treatments with NiCl_2_ were performed by exposing embryos to 0.5 mM of chemical. All treatments were carried out from 30 min to 48 h post-fertilization.

### Cloning of *alk3/6* and *chordin* cDNAs

*Pl-alk3/6*: Degenerated primers were derived from an alignment of the kinase domain from Activin Like Kinase (ALK) Type I receptors. *Pl-chordin*: Degenerated primers were derived from an alignment of the conserved first von Willebrand factor type C of vertebrate Chordin sequences. These primers were used to amplify partial cDNA fragments for both genes using RT PCR and a cDNA mix from mixed embryonic stages. The Primers used to amplify *alk3/6* were as follows: Alk3/6 F1, 5′-GCNTTYATHGCNGCNGAYATHCC-3′, which is derived from the peptide sequence AFIAADIP; Alk3/6 R1, 5′-CATRTCNGCDATCATRCANGTNCC-3′, which is derived from the peptide sequence GTCMIADM. The Primers used to amplify *chordin* were: Chordin F1, 5′-TGYACNTTYGGNGCNGAYTTYTA-3′, which is derived from the peptide sequence CTFGADFY; Chordin R1, 5′-GGRCANGTYTTRCARCARAANCC-3′, which is derived from the peptide sequence GFCCKTCP. PCR fragments for *alk3/6* and *chordin* were subsequently used as probes to screen a pluteus stage cDNA library, and the largest inserts were entirely sequenced on both strands.

The accession numbers of *alk3/6*, *chordin*, and *Smad1/5/8* are, respectively, FJ976181, FJ976182, and FJ976183.

### Site-Directed Mutagenesis and Construction of Expression Plasmids

To make pCS2 Alk4/5/7-Q265D, the CAG codon encoding Glutamine in position 265 of pBS Alk4/5/7 was mutated to GAT. To make pCS2 Alk3/6-Q230D the CAA codon encoding Glutamine in position 230 of pBS Alk3/6 was mutated to GAT. Both constructions were obtained by PCR splicing using two successive rounds of PCR.

Oligonucleotides used for the pCS2-Alk4/5/7-Q265D are:

Alk4/5/7-Cla-ATG, 5′-ACCATCGATACCATGGCATTGGAACGTGCT-3′;

Alk4/5/7-TAG-Xho, 5′-AGGCTCGAGCTACATCTGTAGTTGAGGACG-3′;

Alk4/5/7-Q265D-Fw, 5′-ACCATCGCTCGAGATATCGTGATTCAG-3′;

Alk4/5/7-Q265D-Rev, 5′-CTGAATCACGATATCTCGAGCGATGGT-3′.

Oligonucleotides used for the pCS2-Alk3/6-Q230D are:

Alk3/6-Cla-ATG, 5′-CGGGATCCACCATGGCGACAGATGTAACACTAACCG-3′;

Alk3/6-TAG-Xho, 5′-CCGCTCGAGCTAAACTTTGAATTCCGTTTCTTG-3′;

Alk3/6-Q230D-Fw, 5′-CCGCTCGAGCTAAACTTTGAATTCCGTTTCTTG-3′;

Alk3/6-Q230D-Rev, 5′-CCTAATCAGCTGAACATCCTTCGCGATAGTGCG-3′.

To make the Alk3/6 mm construct, an oligonucleotide containing nine mismatches in the sequence recognized by the morpholino was used to amplify the coding sequence. The sequence of this oligo is:

5′-AGGGGATCCACCATGGCTACGGACGTCACGTTGACCGGACGAAAA-3′,

(mismatches underlined).

Oligonucleotides for making the pCS2 *chordin* construct are:

Chd-Xho-ATG, 5′ AGGCTCGAGACCATGTACCGTGTCGTGATTTATAC-3′;

Chd-Xba-TGA, 5′ TGCTCTAGACTATGAAAGCTTCTCTTTCCTTC-3′.

All PCR reactions were made using the Pfx DNA polymerase and the constructs were verified by sequencing.

### RNA and Morpholino Injections

For overexpression experiments, capped mRNA were synthesized from the pCS2 templates constructs [75] linearized with Not1 using the SP6 mMessage mMachine kit (Ambion). After synthesis, the capped RNA were purified on Sephadex G50 columns and quantitated by spectrophotometry. Synthesis of capped mRNA coding for Nodal and BMP2/4 is described in Duboc et al. (2004) [16]. RNAs were mixed with Tetramethyl Rhodamine Dextran (10,000 MW) or Texas Red Dextran (70,000 MW) or Fluoresceinated Dextran (70,000 MW) at 5 mg/ml and injected in the concentration range 500–800 µg/ml for *nodal* and *alk3/6QD* and in the range 1–1.5 mg/ml for *chordin*. Morpholino antisense oligonucleotides were obtained from GeneTools LLC (Eugene, OR). The sequence of the morpholino against *alk3/6* is 5′-TAGTGTTACATCTGTCGCCATATTC-3′, in which the three underlined bases are complementary to the initiation ATG codon of the transcript. The sequences of the *chordin* morpholinos used are:

Mo1 *chordin*: 5′-GGTATAAATCACGACACGGTACATG-3′; Mo2 *chordin*: CGAAGATAAAAACTTCCAAGGTGTC. Injection of Mo1 *chordin* morpholino caused absolutely no toxicity even when high doses (up to 2 mM) were used while the Mo2 *chordin* started to cause non-specific defects when injected at concentrations above 0.8 mM. As a control, we used a morpholino directed against the hatching enzyme gene: 5′-GCAATATCAAGCCAGAATTCGCCAT-3′. Embryos injected with this morpholino at 1 mM developed into normal pluteus larvae. The *nodal*, *BMP2/4*, and *Alk4/5/7* morpholinos are described in Duboc et al. (2004) [16] and Range et al. (2007) [13]. Morpholinos oligonucleotides were dissolved in sterile water and injected at the one-cell stage together with Tetramethyl Rhodamine Dextran (10,000 MW) at 5 mg/ml. For each morpholino a dose-response curve was obtained and a concentration at which it did not elicit non-specific defect was chosen. Approximately 2–4 pl of oligonucleotide solution at 0.5 mM for Mo-*nodal*1, 0.8–0.6 mM for Mo-*alk3/6*, 1–1.5 mM for Mo-1 *chordin*, and 0.4–0.6 mM for Mo2-*chordin* were used in the experiments described here. For each experiment about 150–200 eggs were injected for morphological observations, and 200–300 for in situ hybridization. All the experiments were repeated at least two to three times and only representative phenotypes observed in a majority of embryos are presented here.

### In Situ Hybridization

In situ hybridization was performed following a protocol adapted from Harland (1991) [76] with antisense RNA probes and staged embryos. The *goosecoid*, *tbx2/3*, *Delta*, and *sm30* probes have been described previously [77],[78]. The *hnf6/onecut* probe was derived from a pBluescript clone obtained by screening a lZAP cDNA library and the *glypican 5* probe from a plasmid library constructed in pSport. Double in situ hybridizations were performed following the procedure of Thisse [79].

### RNA Extraction and Northern Blotting

Total RNA from staged embryos was extracted by the method of Chomczynski and Sacchi (1987) [80]. Samples of total RNA (20 µg per lane) were fractioned on 1% agarose gel containing 0.66 M formaldehyde and transferred to membrane by standard methods (Sambrook et al. 1989) [81].

### Immunostaining

The antibody we used is an anti-phospho-Smad1/5/8 from Cell Signaling (Ref 9511) raised against a synthetic phosphopeptide corresponding to residues around Ser463/465 contained in the motif SSVS of human Smad5. In *S.purpuratus*, both Smad1/5/8 and Smad2/3 share the motif SSVS, but in *P.lividus*, only Smad1/5/8 contains the motif (SSVS), the corresponding motif in *P. lividus* Smad2/3 being SSMS. This antibody specifically recognizes Smad1/5/8 in *P. lividus* (see Figures 5, 6), but it is predicted to recognize both Smad1/5/8 and Smad 2/3 in *S.purpuratus*. Embryos were fixed in paraformaldehyde 4% in MFSW for 15 min, then briefly permeabilized with methanol. Embryos were rinsed with PBST, then PBST-BSA 2% four times and incubated overnight a +4°C with the primary antibody (anti-phospho-Smad1/5/8 Cell Signaling Ref 9511) diluted in PBST supplemented with 2% BSA. Embryos were then washed six times with PBST-BSA 2%, then the secondary antibody diluted in PBST-BSA 2% was added to the embryos. In all cases the antibody was incubated overnight at +4°C. For immunofluorescence, the secondary antibody was washed six times with PBST. Two last rinses were made with PBST-Glycerol 25% and 50%, respectively. Embryos were mounted in a drop of the Citifluor anti-bleaching mounting medium, then observed under a conventional fluorescence microscope or with a confocal microscope. For Alkaline phosphatase revelation, two rinses were made with PBST following the secondary antibody incubation, then two with TBST. Embryos were then washed twice with the alkaline phosphatase buffer supplemented with Tween 0.1%, then staining was performed either with NBT and BCIP as substrates at the final concentration of 50 mM each, or embryos were washed twice with Tris 100 mM pH 8.2 and stained using a FastRed as substrate in Tris100 mM pH 8.2. In both cases staining was stopped by four rinses with PBST+EDTA 5 mM, then two rinses with PBST 25% Glycérol and 50% Glycerol. Embryos were then mounted and observed with a DIC microscope. In the case of the combined immunostaining and in situ hybridization, the immunostaining was performed first as described above. Staining was stopped with four rinses with PBST, then embryos were fixed 1 h in paraformaldehyde 4% in MFSW before proceeding with in situ hybridization.

## Supporting Information

Figure S1 **Alk4/5/7QD is a constitutively active Nodal receptor.** (A) Overexpression of *alk4/5/7QD* induces the same phenotypes as *nodal* overexpression or nickel treatments and ventralizes the ectoderm of sea urchin embryos. Embryos injected with the *alk4/5/7QD* mRNA (ii, iii) are radialized: they contain ectopic spicule rudiments (black arrows) and develop with a proboscis at the animal pole (white arrow) that is typical of embryos radialized by *nodal* overexpression (iv) or nickel treatment (v). (B) Embryos injected with the *alk4/5/7QD* mRNA display a radial expression of ventral marker genes such as *goosecoid* (ii) and *brachyury* (unpublished data). In contrast, inhibition of Alk4/5/7 function using morpholinos abolishes the expression of *goosecoid* (iii, iv). In these embryos, the ciliary band marker gene *hnf6* is expressed throughout the whole ectoderm (v, vi). (C) Partial rescue of the D/V axis of *nodal* Morpholino by injection of the activated Nodal receptor into a vegetal blastomere at the eight-cell stage requires BMP2/4. The scheme of the experiment is depicted (see Figure 1 for the results of animal blastomere injections). (D) Embryos resulting from injection of *alk4/5/7QD* into a vegetal blastomere (i, ii, v, vi) display a significant but partial rescue of D/V polarity. Remarkably, the dorsal region and the antero-lateral arms of the pluteus larva formed normally in these embryos. In contrast, the animal region of the vegetally injected embryos retained the morphology of the Nodal morpholino injected embryos, the oral arms did not form, the mouth did not open, and the ectoderm of this region differentiated into a thick epithelium that surrounded the straight archenteron. Injection of *alk4/5/7QD* mRNA in a vegetal blastomere at the eight-cell stage fails to restore any D/V polarity of embryos previously injected with the *nodal* and BMP2/4 Morpholinos (iii, iv, vii, viii). These embryos never elongated and looked like BMP2/4 morpholino injected embryos (see Figure 1). Note that all the cells that received the *alk4/5/7QD* mRNA are found, respectively, in the ventral vegetal ectoderm and ventral endoderm (viii). In these embryos all cells received the *nodal* morpholino, as they display the RLDX red fluorescence (vii).
(7.23 MB TIF)

Figure S2 **Structure of the *Paracentrotus lividus* Alk4/5/7 and Alk3/6 type I receptors.** (A) Structure of the Alk4/5/7 type I receptor. The positions of the transmembrane domain, GS box, and L45 loop are indicated. (B) Alignment of *Paracentrotus lividus* Alk4/5/7 and Alk3/6 protein sequences with various type I Alk receptor sequences. Boxes above the sequences correspond to the different domains of the protein. The color code used in these boxes is identical to the one used above. Shaded names correspond to *Paracentrotus lividus* receptor sequences. Conservation is low in the N-terminal region of the protein, except for a pattern of cysteines corresponding to the extra-cellular ligand binding domain. Conservation is high within the kinase domain, starting from the GS box. The percentage of identity between the sea urchin Alk3/6 and the human Alk6 over this region reaches 66%, a value comparable to the percentage of identity between *Drosophila* Thickveins and human Alk6 (65%). Dm, *Drosophila melanogaster*; Hs, *Homo sapiens*; Pl, *Paracentrotus lividus*; Sp, *Strongylocentrotus purpuratus*.
(1.06 MB TIF)

Figure S3 **Comparisons of the L45 loop region between different type I Alk receptors.** There are three different consensuses for the L45 loop sequences defining three subgroups of Alk type I receptors: the Alk1/2-Sax and the Alk3/6-Tkv group bind preferentially BMP ligands and activate the Smad1/5/8 factors. Members of the Alk4/5/7-Babo group bind preferentially Nodal, Activin, Univin/Vg1, and TGF-β ligands and activate the Smad2/3 factors. The L45 loop sequence in the sea urchin Alk3/6 protein is highly similar to that of the vertebrate Alk3 and Alk6 proteins as well as to that of the *Drosophila* Thickveins receptor. (B) Alignment of the GS Box region from various type I Alk receptors. The GS box is located on the N-terminal part of the serine-threonine kinase domain of type I Alk receptors. The position of the glutamine residue that was mutated in the *alk4/5/7QD* and *alk3/6QD* constructions used in this study is indicated. Shaded names indicate the two sea urchin sequences used in this study.
(2.34 MB TIF)

Figure S4 **Comparison of *Paracentrotus lividus* Chordin with different Chordin protein sequences from *Nematostella vectensis* (Nv), *Drosophila melanogaster* (Dm), and vertebrate sequences.**
(2.86 MB TIF)

Figure S5 **The sea urchin Chordin protein is a strong BMP antagonist when overexpressed in zebrafish.** (A, B) control embryos at the tailbud stage (A) or at 24 h after fertilization. (C, D) Embryos injected with the sea urchin *chordin* mRNA. At the tail bud stage, *chordin* injected embryos develop with an ovoid shape typical of dorsalized embryos. Indeed, at 24 h these embryos display a strong ventralized phenotype as indicated by the presence of ectopic notochords and radial somites. This phenotype is identical to the *swirl* mutant phenotype that results from disruption of the *bmp2b* gene [44].
(4.24 MB TIF)

## Abbreviations

D/V: dorsal-ventral
