# Supplementary figures and images for: Patterning of the Dorsal-Ventral Axis in Echinoderms: Insights into the Evolution of the BMP-Chordin Signaling Network
Source: PLoS Biol. 2009 Nov 24;7(11):e1000248. doi: 10.1371/journal.pbio.1000248 (PMC2772021; doi:10.1371/journal.pbio.1000248)

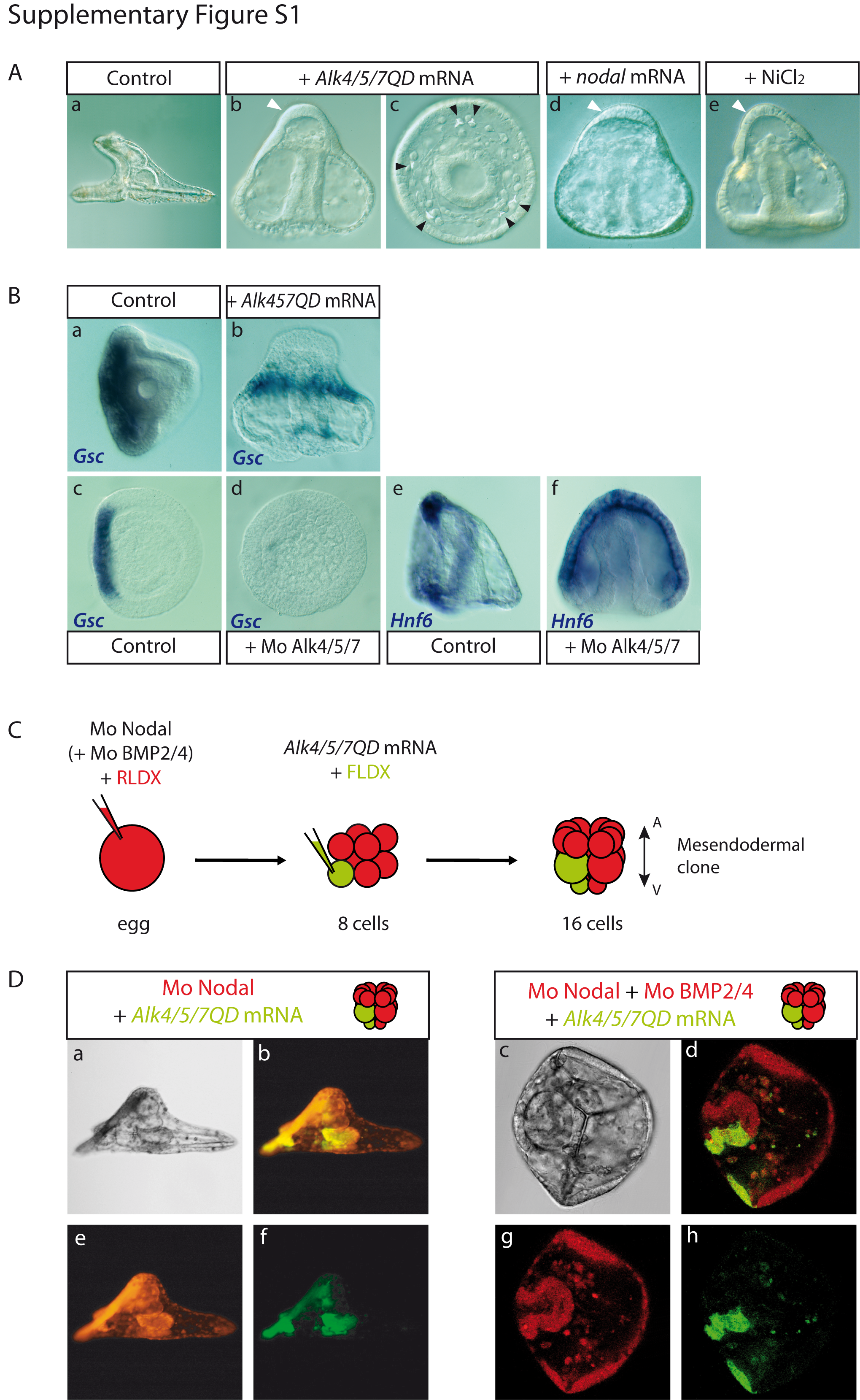

Supplement: Figure S1 — Alk4/5/7QD is a constitutively active Nodal receptor. (A) Overexpression of alk4/5/7QD induces the same phenotypes as nodal overexpression or nickel treatments and ventralizes the ectoderm of sea urchin embryos. Embryos injected with the alk4/5/7QD mRNA (ii, iii) are radialized: they contain ectopic spicule rudiments (black arrows) and develop with a proboscis at the animal pole (white arrow) that is typical of embryos radialized by nodal overexpression (iv) or nickel treatment (v). (B) Embryos injected with the alk4/5/7QD mRNA display a radial expression of ventral marker genes such as goosecoid (ii) and brachyury (unpublished data). In contrast, inhibition of Alk4/5/7 function using morpholinos abolishes the expression of goosecoid (iii, iv). In these embryos, the ciliary band marker gene hnf6 is expressed throughout the whole ectoderm (v, vi). (C) Partial rescue of the D/V axis of nodal Morpholino by injection of the activated Nodal receptor into a vegetal blastomere at the eight-cell stage requires BMP2/4. The scheme of the experiment is depicted (see Figure 1 for the results of animal blastomere injections). (D) Embryos resulting from injection of alk4/5/7QD into a vegetal blastomere (i, ii, v, vi) display a significant but partial rescue of D/V polarity. Remarkably, the dorsal region and the antero-lateral arms of the pluteus larva formed normally in these embryos. In contrast, the animal region of the vegetally injected embryos retained the morphology of the Nodal morpholino injected embryos, the oral arms did not form, the mouth did not open, and the ectoderm of this region differentiated into a thick epithelium that surrounded the straight archenteron. Injection of alk4/5/7QD mRNA in a vegetal blastomere at the eight-cell stage fails to restore any D/V polarity of embryos previously injected with the nodal and BMP2/4 Morpholinos (iii, iv, vii, viii). These embryos never elongated and looked like BMP2/4 morpholino injected embryos (see Figure 1). Note that a [file pbio.1000248.s001.tif]

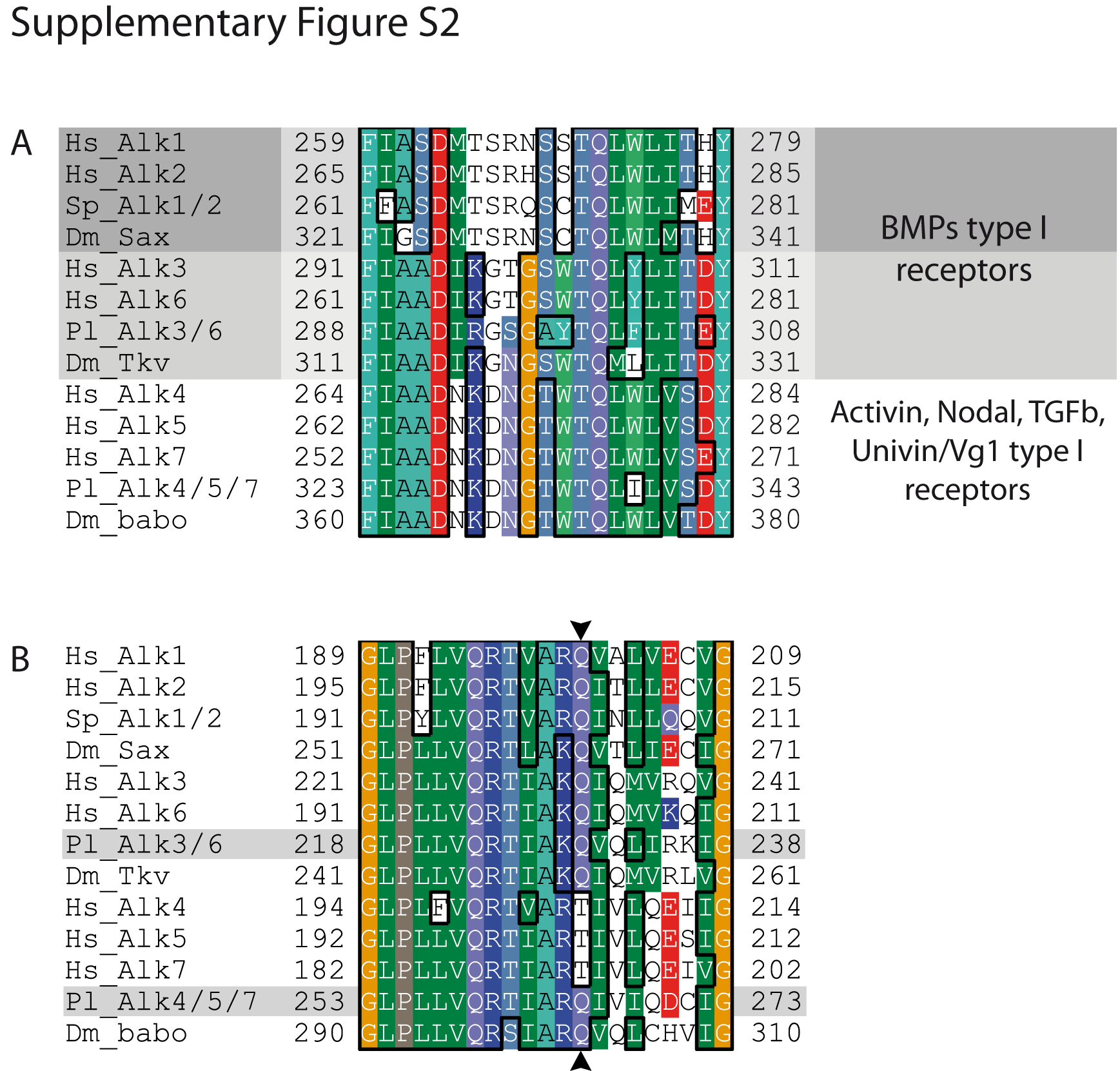

Supplement: Figure S2 — Structure of the Paracentrotus lividus Alk4/5/7 and Alk3/6 type I receptors. (A) Structure of the Alk4/5/7 type I receptor. The positions of the transmembrane domain, GS box, and L45 loop are indicated. (B) Alignment of Paracentrotus lividus Alk4/5/7 and Alk3/6 protein sequences with various type I Alk receptor sequences. Boxes above the sequences correspond to the different domains of the protein. The color code used in these boxes is identical to the one used above. Shaded names correspond to Paracentrotus lividus receptor sequences. Conservation is low in the N-terminal region of the protein, except for a pattern of cysteines corresponding to the extra-cellular ligand binding domain. Conservation is high within the kinase domain, starting from the GS box. The percentage of identity between the sea urchin Alk3/6 and the human Alk6 over this region reaches 66%, a value comparable to the percentage of identity between Drosophila Thickveins and human Alk6 (65%). Dm, Drosophila melanogaster; Hs, Homo sapiens; Pl, Paracentrotus lividus; Sp, Strongylocentrotus purpuratus. (1.06 MB TIF) [file pbio.1000248.s002.tif]

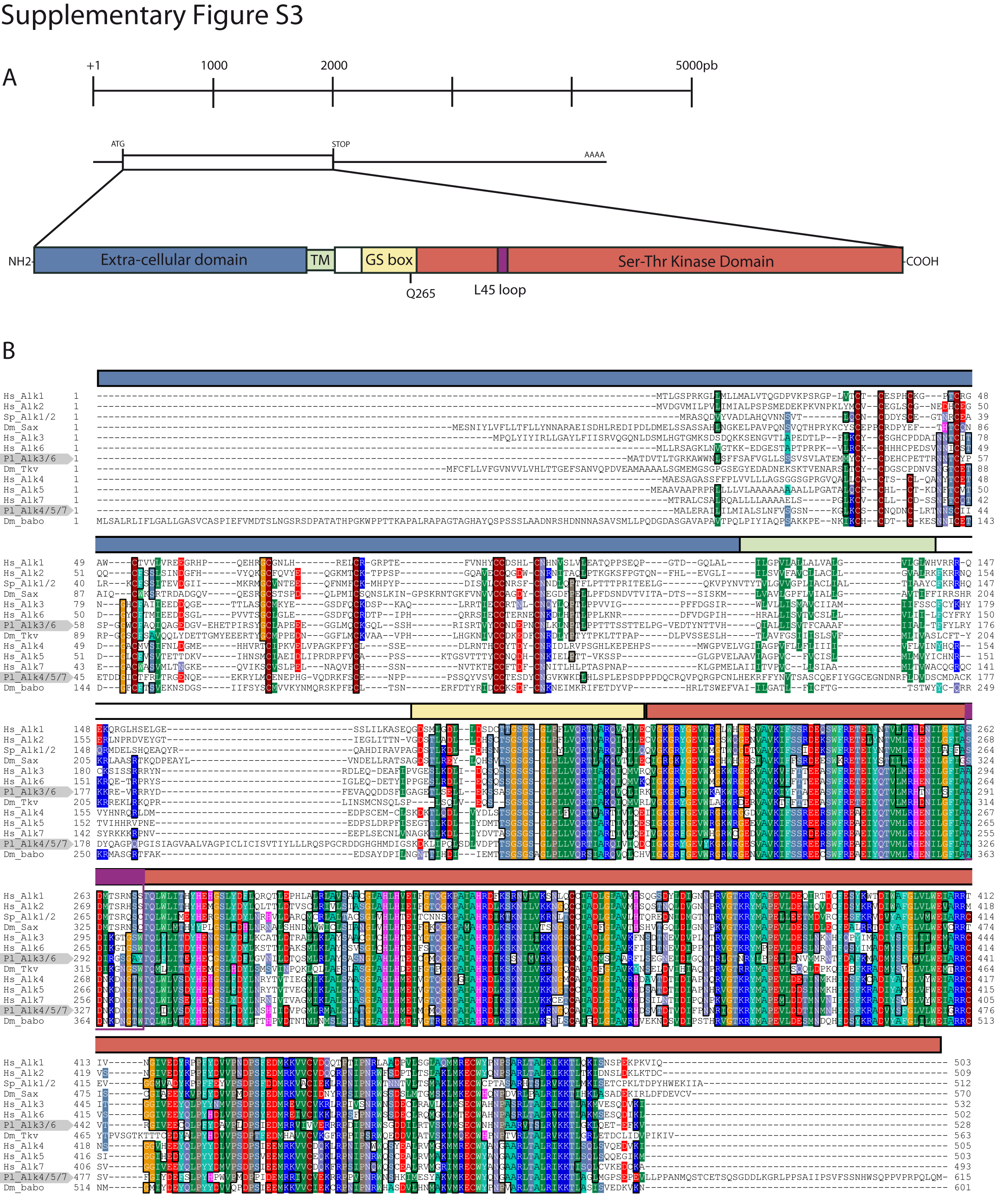

Supplement: Figure S3 — Comparisons of the L45 loop region between different type I Alk receptors. There are three different consensuses for the L45 loop sequences defining three subgroups of Alk type I receptors: the Alk1/2-Sax and the Alk3/6-Tkv group bind preferentially BMP ligands and activate the Smad1/5/8 factors. Members of the Alk4/5/7-Babo group bind preferentially Nodal, Activin, Univin/Vg1, and TGF-β ligands and activate the Smad2/3 factors. The L45 loop sequence in the sea urchin Alk3/6 protein is highly similar to that of the vertebrate Alk3 and Alk6 proteins as well as to that of the Drosophila Thickveins receptor. (B) Alignment of the GS Box region from various type I Alk receptors. The GS box is located on the N-terminal part of the serine-threonine kinase domain of type I Alk receptors. The position of the glutamine residue that was mutated in the alk4/5/7QD and alk3/6QD constructions used in this study is indicated. Shaded names indicate the two sea urchin sequences used in this study. (2.34 MB TIF) [file pbio.1000248.s003.tif]

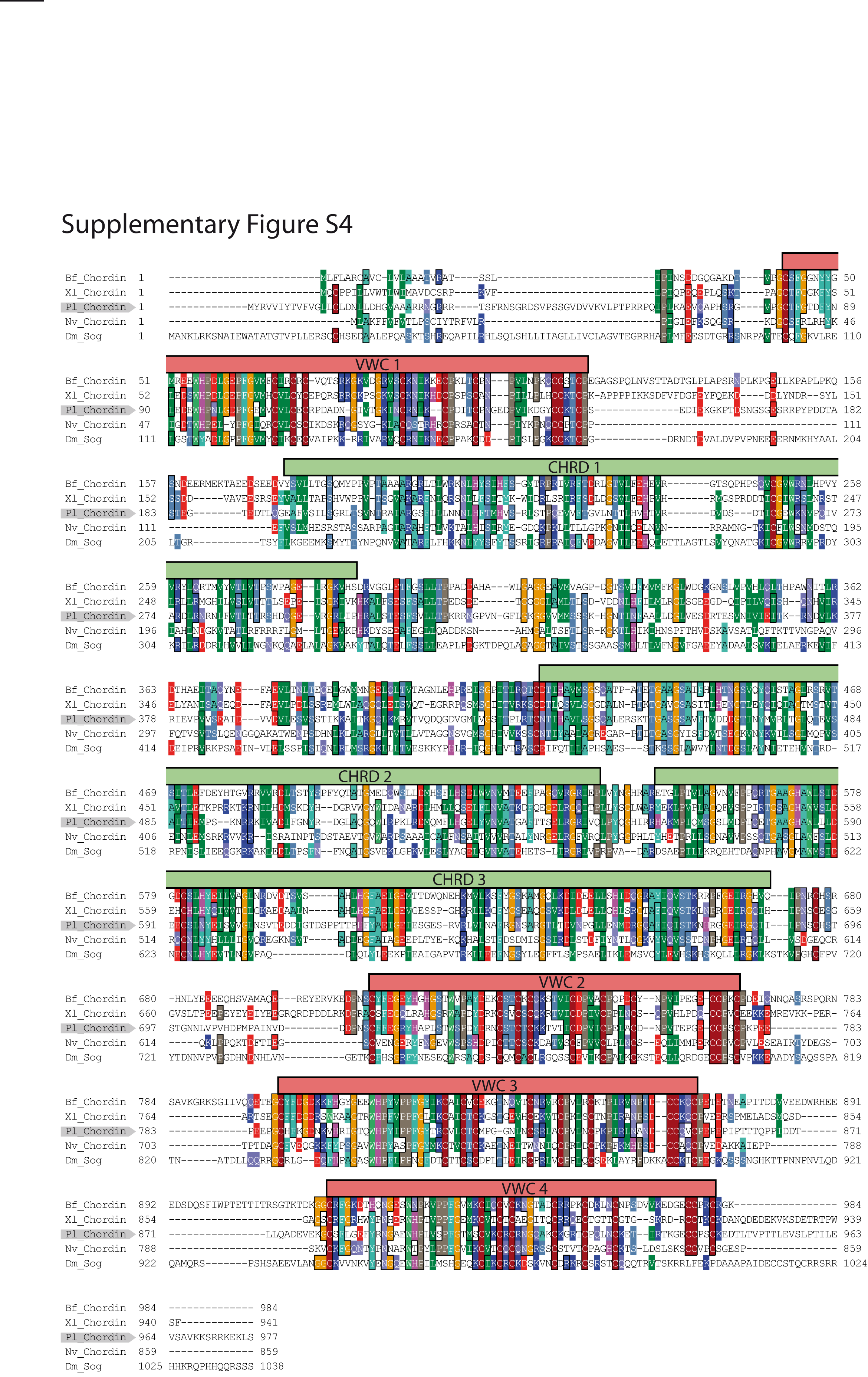

Supplement: Figure S4 — Comparison of Paracentrotus lividus Chordin with different Chordin protein sequences from Nematostella vectensis (Nv), Drosophila melanogaster (Dm), and vertebrate sequences. (2.86 MB TIF) [file pbio.1000248.s004.tif]

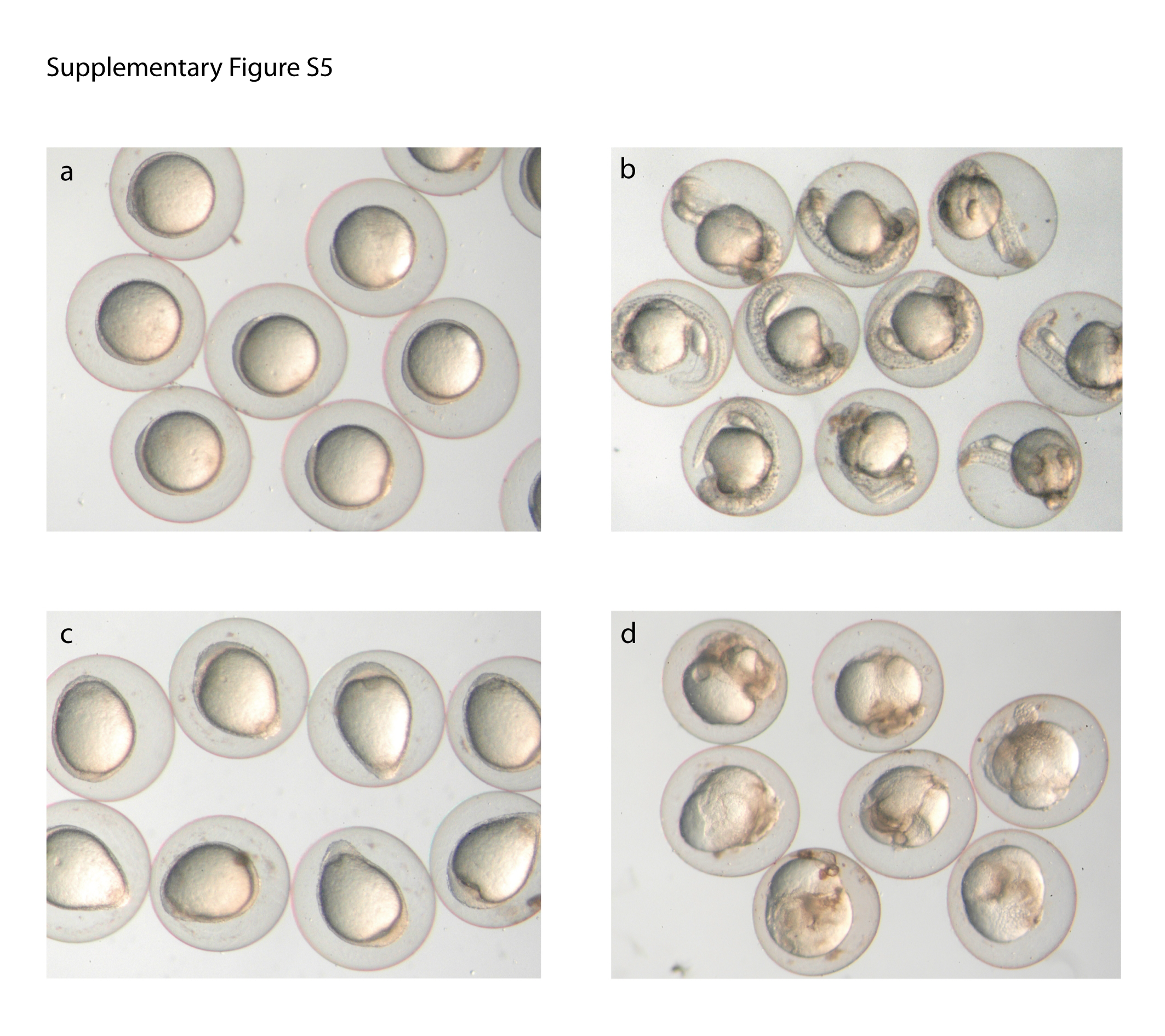

Supplement: Figure S5 — The sea urchin Chordin protein is a strong BMP antagonist when overexpressed in zebrafish. (A, B) control embryos at the tailbud stage (A) or at 24 h after fertilization. (C, D) Embryos injected with the sea urchin chordin mRNA. At the tail bud stage, chordin injected embryos develop with an ovoid shape typical of dorsalized embryos. Indeed, at 24 h these embryos display a strong ventralized phenotype as indicated by the presence of ectopic notochords and radial somites. This phenotype is identical to the swirl mutant phenotype that results from disruption of the bmp2b gene [44]. (4.24 MB TIF) [file pbio.1000248.s005.tif]
